# 1,4-Disubstituted 1*H*-1,2,3-Triazole Containing Peptidotriazolamers: A New Class of Peptidomimetics With Interesting Foldamer Properties

**DOI:** 10.3389/fchem.2019.00155

**Published:** 2019-03-26

**Authors:** David C. Schröder, Oliver Kracker, Tanja Fröhr, Jerzy Góra, Michał Jewginski, Anke Nieß, Iris Antes, Rafał Latajka, Antoine Marion, Norbert Sewald

**Affiliations:** ^1^Organic and Bioorganic Chemistry, Department of Chemistry, Bielefeld University, Bielefeld, Germany; ^2^Department of Bioorganic Chemistry, Wrocław University of Science and Technology, Wrocław, Poland; ^3^Center for Integrated Protein Science, TUM School of Life Sciences, TU Munich, Freising, Germany; ^4^Department of Chemistry, Middle East Technical University, Ankara, Turkey

**Keywords:** peptidotriazolamers, 1, 4-disubstituted 1*H*-1, 2, 3-triazole, peptide bond isoster, foldamer, molecular dynamic simulations

## Abstract

Peptidotriazolamers are hybrid foldamers with features of peptides and triazolamers, containing alternation of amide bonds and 1,4-disubstituted 1*H*-1,2,3-triazoles with conservation of the amino acid side chains. We report on the synthesis of a new class of peptidomimetics, containing 1,4-disubstituted 1*H*-1,2,3-triazoles in alternation with amide bonds and the elucidation of their conformational properties in solution. Based on enantiomerically pure propargylamines bearing the stereogenic center in the propargylic position and α-azido esters, building blocks were obtained by copper-catalyzed azide-alkyne cycloaddition. With these building blocks the peptidotriazolamers were readily available by solution phase synthesis. A panel of homo- and heterochiral tetramers, hexamers, and heptamers was synthesized and the heptamer Boc-Ala-Val-Ψ[4Tz]Phe-LeuΨ[4Tz]Phe-LeuΨ[4Tz]Val-OAll as well as an heterochiral and a Gly-containing equivalent were structurally characterized by NMR-based molecular dynamics simulations using a specifically tailored force field to determine their conformational and solvation properties. All three variants adopt a compact folded conformation in DMSO as well as in water. In addition to the heptamers we predicted the conformational behavior of similar longer oligomers i.e., Boc-Ala-(AlaΨ[4Tz]Ala)_6_-OAll as well as Boc-Ala-(d-AlaΨ[4Tz]Ala)_6_-OAll and Boc-Ala-(GlyΨ[4Tz]Ala)_6_-OAll. Our calculations predict a clear secondary structure of the first two molecules in DMSO that collapses in water due to the hydrophobic character of the side chains. The homochiral compound folds into a regular helical structure and the heterochiral one shows a twisted “S”-shape, while the Gly variant exhibits no clear secondary structure.

## Introduction

**Figure 1:**
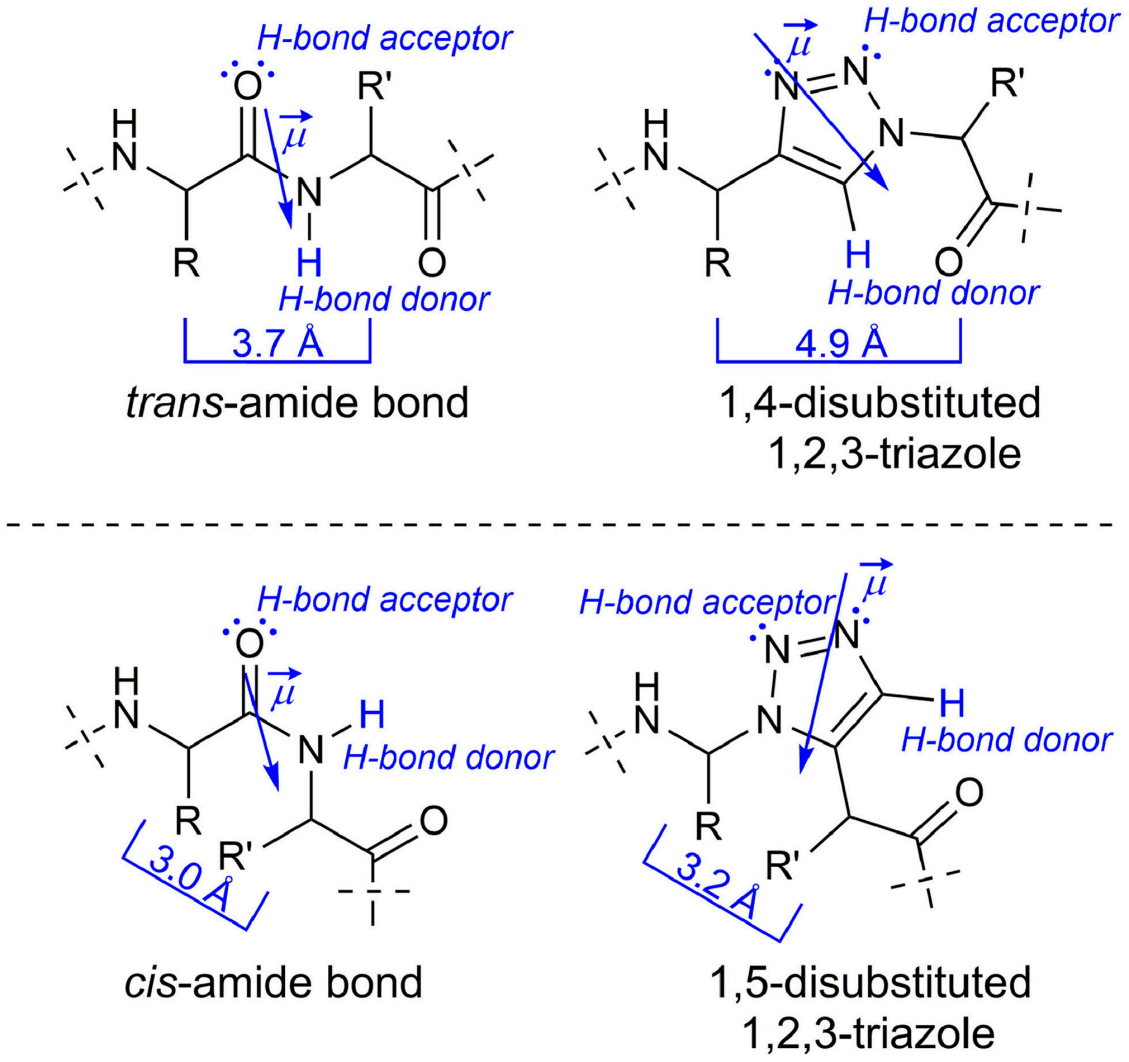
Comparison of a native peptide bond with 1*H*-1,2,3-triazoles as amide bond isoster.

Since the discovery of the Cu^I^ catalyzed azide-alkyne cycloaddition (CuAAC) by the groups of Meldal (Tornøe et al., 2002) and Sharpless (Rostovtsev et al., 2002), this so-called “click”-reaction proved to be suitable for a broad number of applications in bioorganic and pharmaceutical chemistry (Kolb and Sharpless, 2003; Bock et al., 2006a; Angell and Burgess, 2007; Gil et al., 2007; Moses and Moorhouse, 2007; Hein et al., 2008; Tron et al., 2008; Nwe and Brechbiel, 2009) The CuAAC is compatible with most functional groups present in biomolecules and the 1,4-disubstituted 1*H*-1,2,3-triazole obtained as reaction product is metabolically inert (Kolb and Sharpless, 2003). Size and dipole moment of the 1,4-disubstituted triazole ring are larger compared to a *trans*-amide bond (Kolb and Sharpless, 2003), but overall physicochemical properties are similar enough to enable these triazoles to act as *trans*-amide mimetics (Figure 1; Brik et al., 2003, 2005; Horne et al., 2004, 2009; Angell and Burgess, 2005, 2007; van Maarseveen et al., 2005; Bock et al., 2006b, 2007; Wang et al., 2006; Appendino et al., 2007; Kim et al., 2007; Lee et al., 2007; Beierle et al., 2009).

In 2005 the counterpart of the CuAAC, the ruthenium(II)-catalyzed azide-alkyne-cycloaddition (RuAAC), yielding selectively 1,5-disubstitued 1*H*-1,2,3-triazoles, was presented by Zhang et al. (2005). The latter can act as *cis*-amide mimetic (Figure 1; Zhang et al., 2005; Pedersen and Abell, 2011).

Several amide bonds within biologically active substances have already been replaced by triazoles. The bio-isosterism has been exemplified by triazole analogs of a matrix metalloprotease inhibitor (Wang et al., 2006), of the immune-stimulating natural compound α-galactosylceramide (Kim et al., 2007; Lee et al., 2007), and of capsaicin in its role as agonist of the vanilloid-receptor TRPV1 (Appendino et al., 2007). Small bivalent peptidomimetics based on triazole formation from amino acid-derived propargylamines and amino acid-derived α-azido acids were shown to adopt turn-like conformations and serve as ligands for the receptor TrkC (Chen et al., 2009).

Further examples are triazole analogs of the peptidic tyrosinase inhibitor *cyclo*-[Pro-Tyr-Pro-Val] (Bock et al., 2006b, 2007), of the histone deacetylase inhibitor apicidin (Horne et al., 2009), the antimitotic cyclodepsipeptide cryptophycin (Nahrwold et al., 2010) and of peptides containing the pharmacophoric residues of somatostatin (Beierle et al., 2009). Moreover, X-ray crystal structure analysis revealed that the triazole ring within an analog of the HIV-1-protease inhibitor amprenavir interacts with the enzyme in the same way as an amide bond in the parent compound (Brik et al., 2003, 2005). Recently Ben Haj Salah et al. gave a good overview on the topic of substituting single or isolated amide moieties by 1*H*-1,2,3-triazoles and showed the influence of isolated triazole-amide-bond substitution as well as two cases with multiple substitutions containing a motif of two triazole replacements separated by one amide bond in two antimicrobial peptides with helical conformations (Ben Haj Salah et al., 2018).

**Figure 2:**
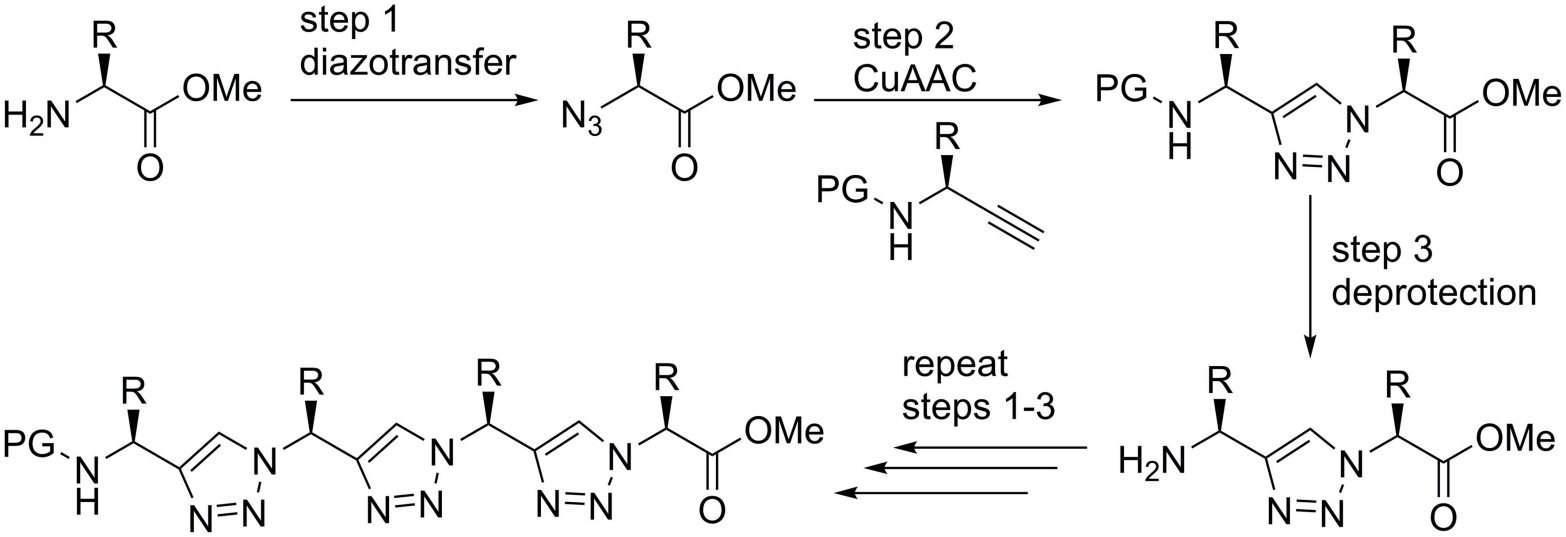
Solution phase synthesis of triazolamers according to Angelo and Arora (2005).

Triazolamers, oligomers of 1,4-disubstituted triazoles connect a series of triazole rings by single C_1_ unit that, at the same time, is a stereogenic center (Angelo and Arora, 2005, 2007; Jochim et al., 2009). The CuAAC can be performed either in solution or on solid phase to provide the 1,4-disubsituted triazoles. Best results are obtained in a sequential protocol when using triflic azide and Cu(II) for the diazo transfer, where the copper was subsequently reduced by ascorbate to bring about the CuAAC (Figure 2; Angelo and Arora, 2005; Beckmann and Wittmann, 2007).

Since the triazole ring is characterized by a large dipole moment it might stabilize discrete conformations by dipole-dipole interactions. The Arora group investigated relatively short oligomers and found out that they are likely to adopt a zigzag conformation reminiscent of β-strands found in peptides. Indeed, the dipole-dipole-interaction of neighboring triazole moieties seems to crucially influence the overall conformation in polar solvents (Angelo and Arora, 2005).

The zigzag triazolamer structure also resembles the oligopyrrolinones described by Smith and Hirschmann (Smith et al., 1992). The axial C^β^ to C^β^ distance between the *i* and *i*+*2* side chain residues in peptide β-strands is 7.2 Å; this distance is roughly 7.9 Å in the zigzag triazolamer. The C^β^ to C^β^ distances in adjacent residues are 5.5 Å in peptide β-strands and a little longer (6.8 Å) in the triazolamer (Angelo and Arora, 2005).

In addition, the group of L. Mindt investigated besides the substitution of isolated peptide bonds by 1,4-disubstituted triazoles also the replacement of multiple adjacent peptide bonds attempting to improve the tumor targeting qualities of bombesin, an amphibious analog of the gastrin releasing peptide (Valverde et al., 2015).

**Figure 3:**
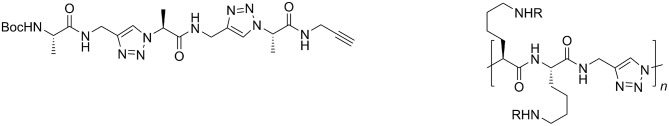
Triazole-based peptidomimetic with unsubstituted propargylamines (left) and “Click”-polyaddition oligomer; bifunctional monomers from amino acid-derived α-azido acids and unsubstituted propargylamine (right).

Peptidotriazole foldamers represent repetitive hybrid structures of peptides, where e.g., every second amide bond is being replaced by a triazole. Such short triazole-based peptide analogs have been obtained from achiral propargylamines and α-amino acid derived α-azido carboxylic acids (Figure 3, left). They were found to self-dimerize and act as organogelators (Ke et al., 2012).

In a similar fashion, the Hecht group synthesized bifunctional monomers from amino acid-derived α-azido acids and propargylamine for “click”-polyaddition (Figure 3, right; Hartwig and Hecht, 2010).

In accordance with recommendations for nomenclature of amide bond replacements we will use Ψ[4Tz] in this publication as a placeholder of a 1,4-disubstituted 1*H*-1,2,3-triazole–i.e., a dipeptide H-Xaa-Yaa-OH is replaced by H-XaaΨ[4Tz]Yaa-OH. In a similar fashion Ψ[5Tz] stands for a 1,5-disubstituted 1*H*-1,2,3-triazole replacement in the same orientation.

Recently we published on 1,5-disubstituted 1,2,3-triazole containing peptidotriazolamers (Kracker et al., 2018). Beside the robust synthetic route it was shown that the homochiral and heterochiral oligomers adopt versatile foldamer conformations. Furthermore, the solid state conformation of the homochiral tetramer Boc-ValΨ[5Tz]Ala-LeuΨ[5Tz]Val-OBzl was in an excellent agreement with an *in silico* conformational analysis, which was performed using a specifically truncated TZLff molecular mechanics force-field parametrization for 1,4- and 1,5-disubstituted peptidotriazolamers that we recently established (Marion et al., 2018).

**Figure 4:**
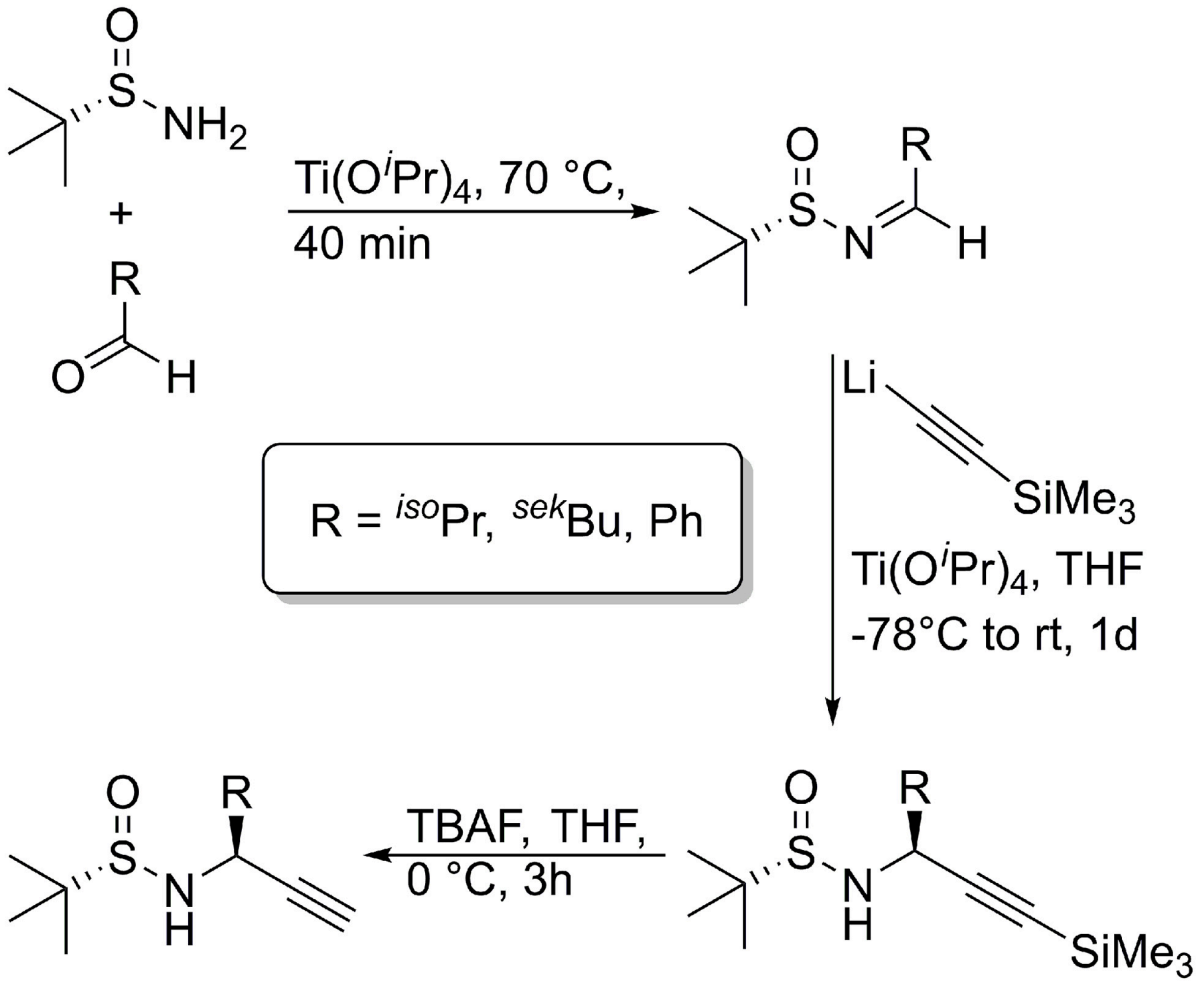
Synthesis of diasteromerically pure *N*-sulfinyl propargylamines, used for the oligomers of this publication.

For the synthesis of the chiral propargylamines, we used the methodology previously described by Ellman et al. using (*R*)- or (*S*)-*tert*-butylsulfinamide as a chiral auxiliary (Patterson and Ellman, 2006). According to the protocol of Ellman et al. the chiral *tert*-butylsulfinamide is prepared by enantioselective oxidation of di-*tert*-butyl disulfide with a chiral catalyst, followed by reaction with lithiumamide (Weix and Ellman, 2005). After formation of the imine using the auxiliary and an aldehyde as starting material, the diastereoselective synthesis of propargylamines is possible by reaction of the *N*-sulfinyl imine with lithium trimethylsilylacetylide (Wünsch et al., 2017). Cleavage of the trimethylsilyl protecting group is achieved with TBAF or AgNO_3_ to provide the *N*-sulfinylpropargylamines with a terminal alkyne moiety. Numerous *tert*-butylsulfinyl protected propargylamines [(*S*)- or (*R*)-Bus-Xaa≡CH] have been prepared in our group, bearing natural amino acid side chains as well as non-amino acid like side chains (Figure 4; Wünsch et al., 2017).

## Results and Discussion

### Synthesis

Inspired by the Arora triazolamers, we embarked on a project directed toward the synthesis and characterization of peptidotriazole foldamers, where both components, the propargylamine and the α-azido carboxylic acids are chiral. The chiral propargylamines were synthesized via the Ellman strategy and the α-azidocarboxylic acids from commercially available α-amino acids by diazotransfer (Yan et al., 2005). Several α-azidocarboxylic acids as well as the corresponding amino acid allyl esters can be synthesized under mild reaction conditions with good to very good yields.

**Figure 5:**
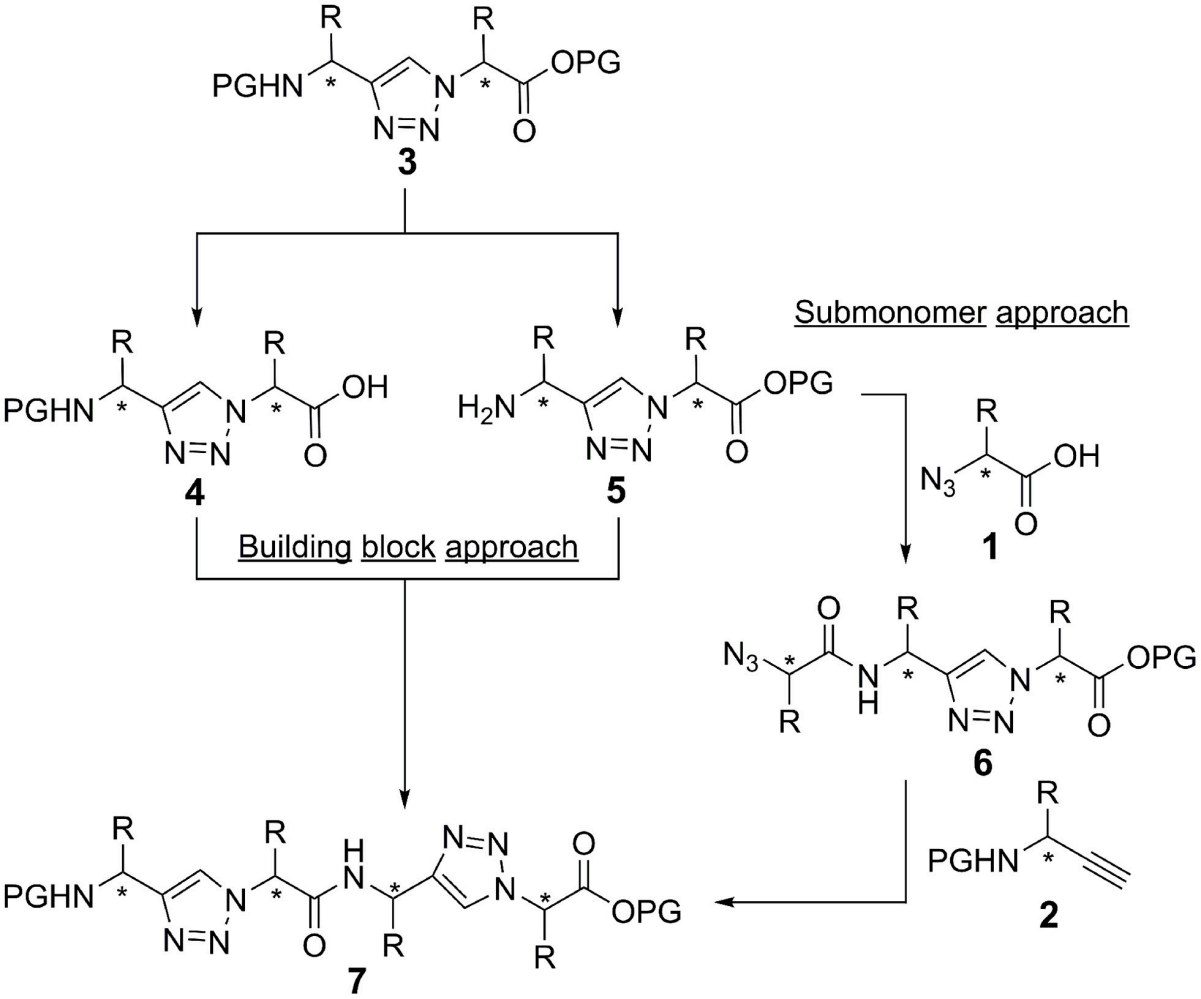
Building block and submonomer approach. Stereogenic centers are labeld with an asterisk.

In principle, there are two strategies for the synthesis of peptidotriazole foldamers: the building block approach (Figure 5), where the triazole is formed from the propargylamine and the azide by CuAAC and then coupled by amide bond formation, and the submonomer approach (Figure 5), where first the α-azido carboxylic acid is coupled and then the *N*-protected propargylamine is reacted by CuAAC.

In the case of activation of the free acid of the triazole-building block **4** (Val side chain), epimerization was observed even upon using TBTU/HOBt/DIPEA or HATU/HOAt/DIPEA as the coupling reagents. Therefore, the submonomer approach was initially chosen for the synthesis of the peptidotriazole foldamers. In this case the α-azido carboxylic acid can be activated with e.g., HATU/HOAt without any epimerization. Eventually it has been shown, that a carbodiimide-mediated preactivation in DCM, without the use of a base, and subsequent coupling with the use of 2,4,6-collidine, effectively prevents epimerization during the coupling of the free acid of the triazole-building block **4** (Kracker et al., 2018). K Ben Haj Salah et al. also used the building block approach for their synthesis of peptaibols analogs containing AibΨ[4Tz]-Xaa dipeptides (Ben Haj Salah et al., 2015).

The *C*-terminal component was protected as allyl ester and the propargylamines were employed as sulfinamide as well as Boc protected derivatives. The *tert*-butylsulfinyl group (Bus) is acid-labile and can be cleaved e.g., by treatment with 4 M HCl in dioxane at room temperature. In addition, it was reported to be cleavable with 50% trifluoroacetic acid in CH_2_Cl_2_ (Lee and Silverman, 2000).

**Figure 6:**
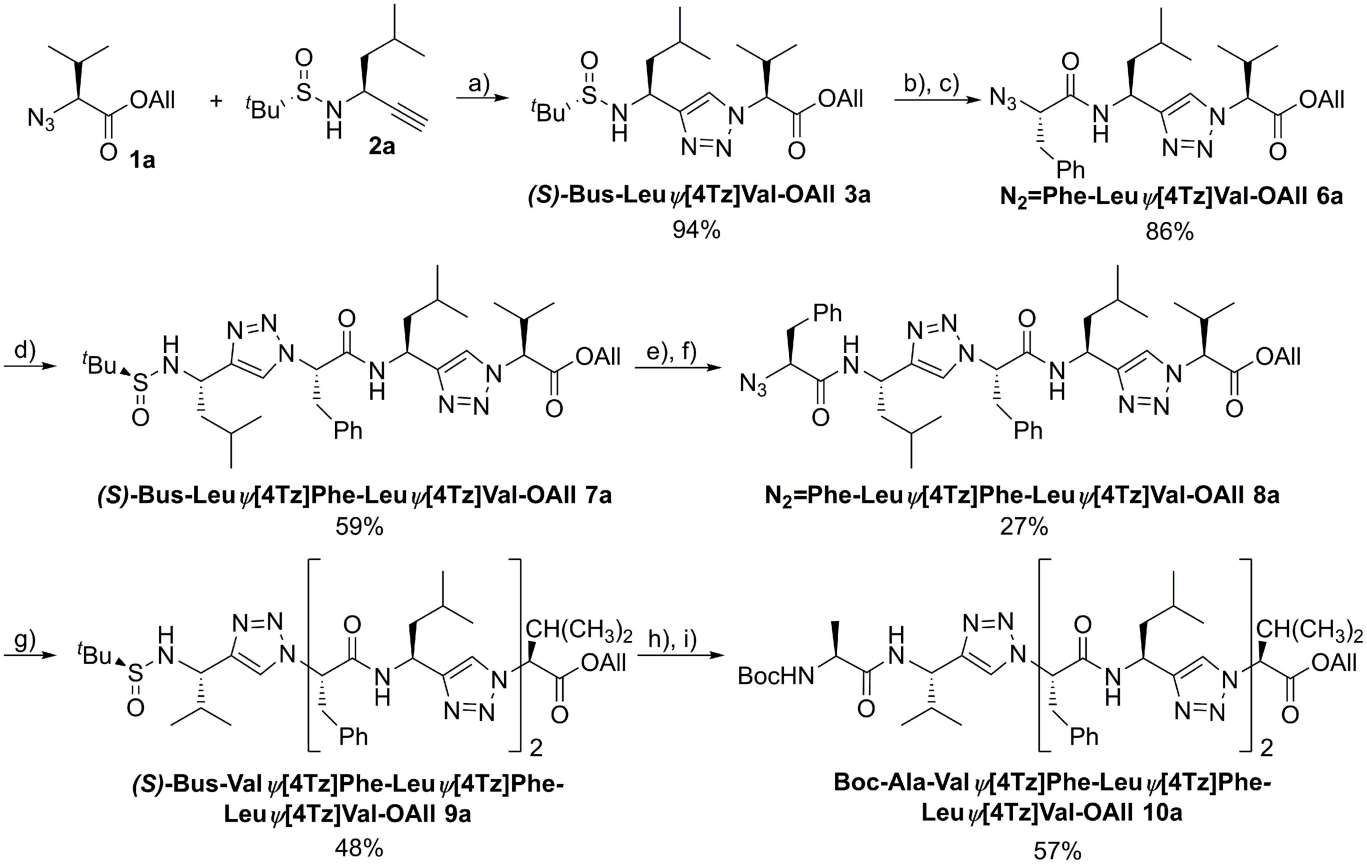
Synthesis of the homochiral peptidotriazole Boc-Ala-ValΨ[4Tz]Phe-LeuΨ[4Tz]Phe-LeuΨ[4Tz]Val-OAll (**10a**). a) CuSO_4_·5H_2_O, sodium ascorbate, H_2_O/DMF, RT, 15 h; b) 4 M HCl in dioxane, MeOH, RT, 2 h; c) N_2_ = Phe-OH, HATU, HOAt, DIPEA, DMF, RT, 15 h; d) (*S*)-Bus-Leu-CH, CuSO_4_·5H_2_O, sodium ascorbate, H_2_O/DMF, RT, 15 h; e) 4 M HCl in dioxane, MeOH, RT, 2 h; f) N_2_ = Phe-OH, HATU, HOAt, DIPEA, DMF, RT, 15 h; g) (*S*)-Bus-Val-CH, CuSO_4_·5H_2_O, sodium ascorbate, H_2_O/DMF, RT, 15 h; h) 4 M HCl in dioxane, MeOH, RT, 2 h; i) Boc-Ala-OH, HATU, HOAt, DIPEA, DMF, RT, 15 h.

An example of the peptidotriazole foldamer synthesis is given in Figure 6. Triazole formation was achieved by CuAAC in the presence of 0.5 eq. of CuSO_4_·5 H_2_O and 1 eq. sodium ascorbate in DMF/water mixtures. Lower amounts of copper salt and reductant led to incomplete conversion, as had been previously observed by others (Angelo and Arora, 2005).

**Figure 7:**
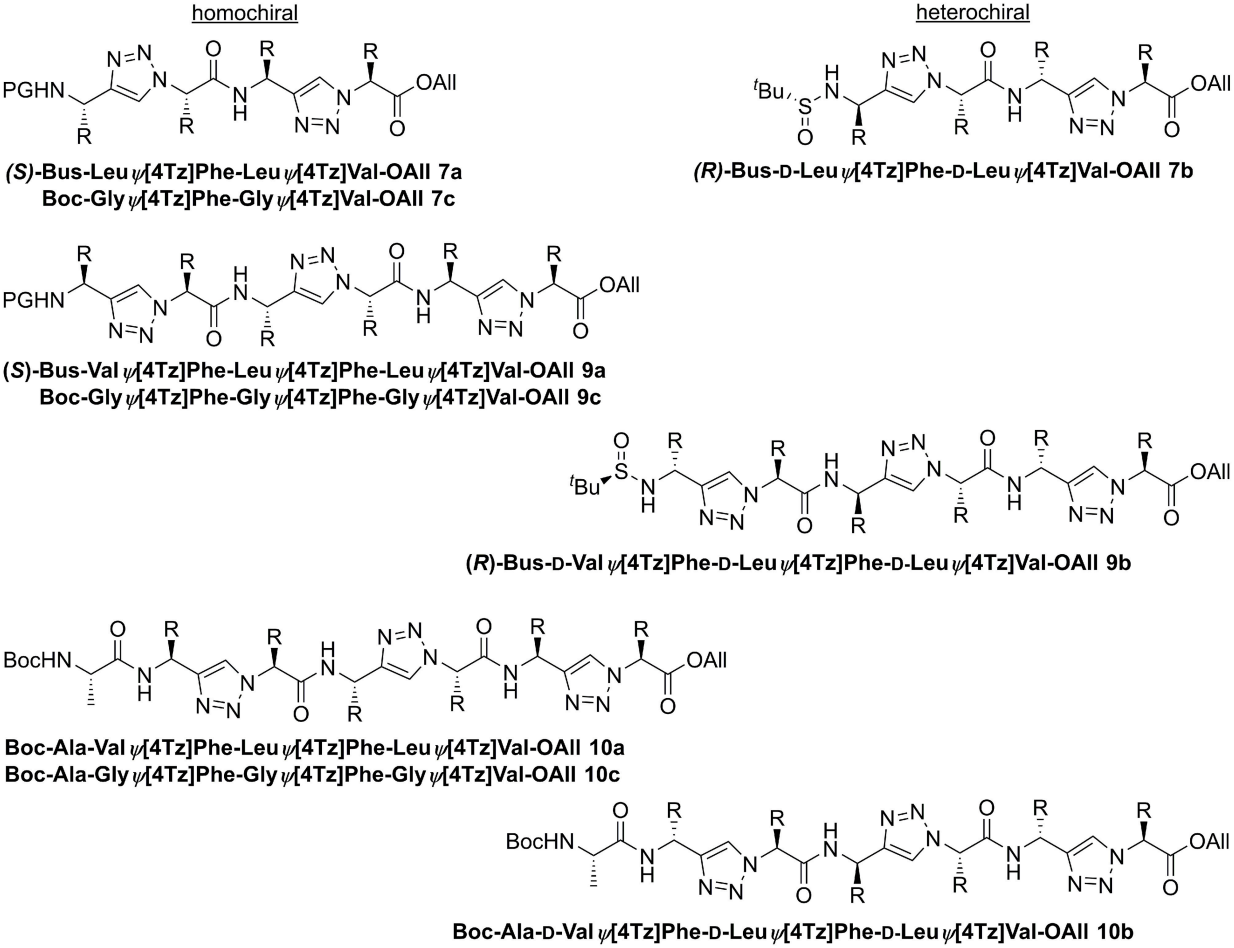
Homo- and heterochiral peptidotriazole tetramers, hexamers, and heptamers.

Several homo- (**7a**, **9a**, **10a**) and heterochiral (**7b**, **9b**, **10b**) peptidotriazole foldamers as well as one where every second amino acid side chain is exchanged by a hydrogen atom (**7c**, **9c**, **10c**), have been obtained (Figure 7). Some peptidotriazole foldamers with *N*-terminal Bus group that had been purified by HPLC with an acetonitrile/water/TFA gradient slowly decomposed in DMSO solution upon standing. Notably, this instability also depends on the sequence. After cleavage of the Bus group and acylation e.g., with Boc-Ala the resulting heptamers were completely stable. The *tert*-butylsulfinyl group had been suggested as a substitute for the Boc group. However, its stability, in particular at elevated temperatures, is not sufficient to qualify Bus as a versatile protecting group. According to the rearrangement suggested for the Ellman auxiliary by Arava et al. (2011) we found out that a fragment (*S*) or (*R*)-BusXaaΨ[4Tz]Yaa-OAll rearranges with a second equivalent to form (*S*) or (*R*)-H-XaaΨ[4Tz]Yaa-OAll and (*tert-*Bu-SO_2_)(*tert*-Bu-S)Xaa Ψ[4Tz]Yaa-OAll.

### Conformational Analysis

**Figure 8:**
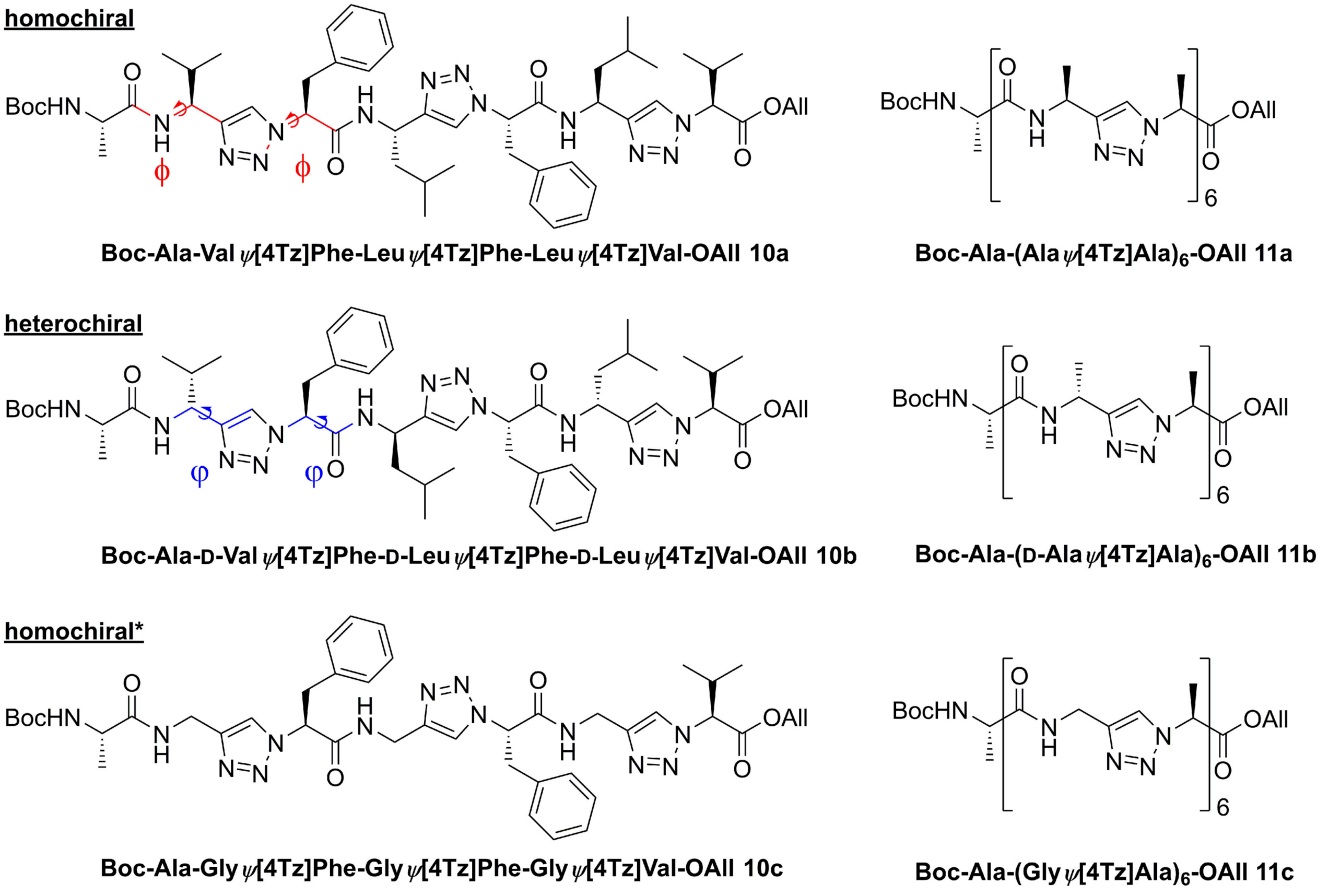
Homo- and heterochiral peptidotriazolamers as representatives for *in silico* structure determination. The definition for the torsion angles ϕ and ψ for these peptidomimetic torsions is annotated by the colored bonds.

We analyzed the conformational preferences of 1,4-disubstituted peptidotriazolamers in solution by means of CD spectroscopy and molecular dynamics (MD) simulations based on the specific molecular mechanics force-field parameterization TZLff (Marion et al., 2018). As representative molecules, we selected compounds **10a**, **10b**, and **10c** (further referred to as homochiral, heterochiral, and homochiral^*^, respectively) to investigate the effect of chirality and of the introduction of glycine-like moieties on the conformational preferences of the peptide mimetics. Schematic representations of the three molecules are given in Figure 8. Therein, we additionally indicate our definition for the backbone conformational degrees of freedom for peptidotriazolamers as the ϕ and ψ torsion angles. In the following, ϕ and ψ notations are used indifferently whether it refers to a standard amino acid or to a triazole-based derivative (Marion et al., 2018).

#### CD Spectroscopy

**Figure 9:**
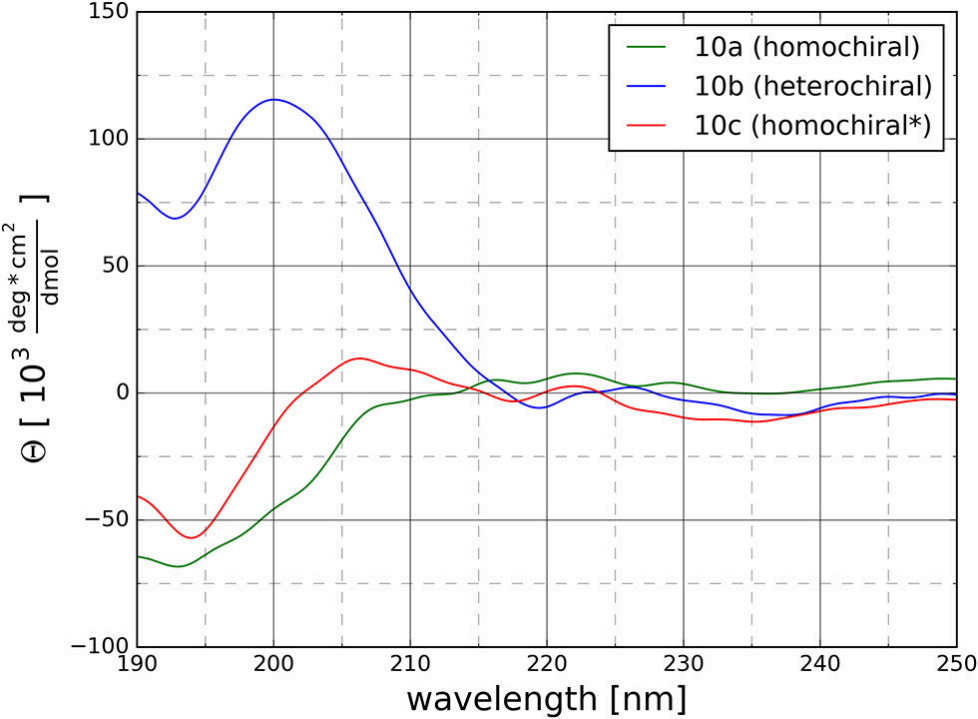
CD Spectra for the homochiral (**10a**), the heterochiral (**10b**), and the homochiral^*^ (**10c**) oligomers measured at 20°C at a concentration of 125 μM in acetonitrile.

CD spectra were recorded to get a first experimental insight into the conformational properties of the three oligomer types. It should be emphasized that the solvent used for the measurements is acetonitrile since DMSO is not applicable due to its strong absorbance below 270 nm. It becomes clear (Figure 9) that the different types of peptidotriazole heptamers have characteristic CD signatures. The heterochiral compound **10b** has a positive CD below 210 nm, while the CD remains negative for the homochiral all-(*S*)-configured **10a**. The CD spectrum of **10c** is somewhat intermediate, with a sign inversion at about 202 nm.

#### Molecular Dynamics

For each of the three representative molecules (**10a-c**), we performed one simulation in DMSO (i.e., the solvent used for NMR measurements) and one simulation in water, as representative of a more biologically relevant environment. The simulations in solution were started using an initial structure derived from an extensive, simulated annealing-based sampling in gas phase during which NMR-derived interproton distance restraints were applied (see section Methodology for details).

Monitoring of the backbone root mean square deviations (RMSD) along the trajectories of each molecule showed that most compounds adopt one main stable conformation in both solvents, with standard deviations ranging from 0.18 to 0.79 Å over the whole trajectory for all six MD trajectories (Supplementary Figure 10). Repetitive bumps in the RMSD time series of **10c** in water show that the molecules adopt at least two stable conformations in water and that our MD simulations sampled them appropriately. Radius of gyration (ROG) time series indicate similar results for all compounds with mean values ranging from 3.5 to 4.0 Å with minor amplitudes of fluctuations after 100 ns (Supplementary Figure 11). Such small values for the ROG of the 1,4-disubstituted peptidotriazolamers are indicative for a compact folded conformation for all three variants. All the time series indicate that the simulations reached convergence after 100 ns at most. We therefore considered the last 100 ns as converged and used this part in each simulation for the following analysis.

**Figure 10:**
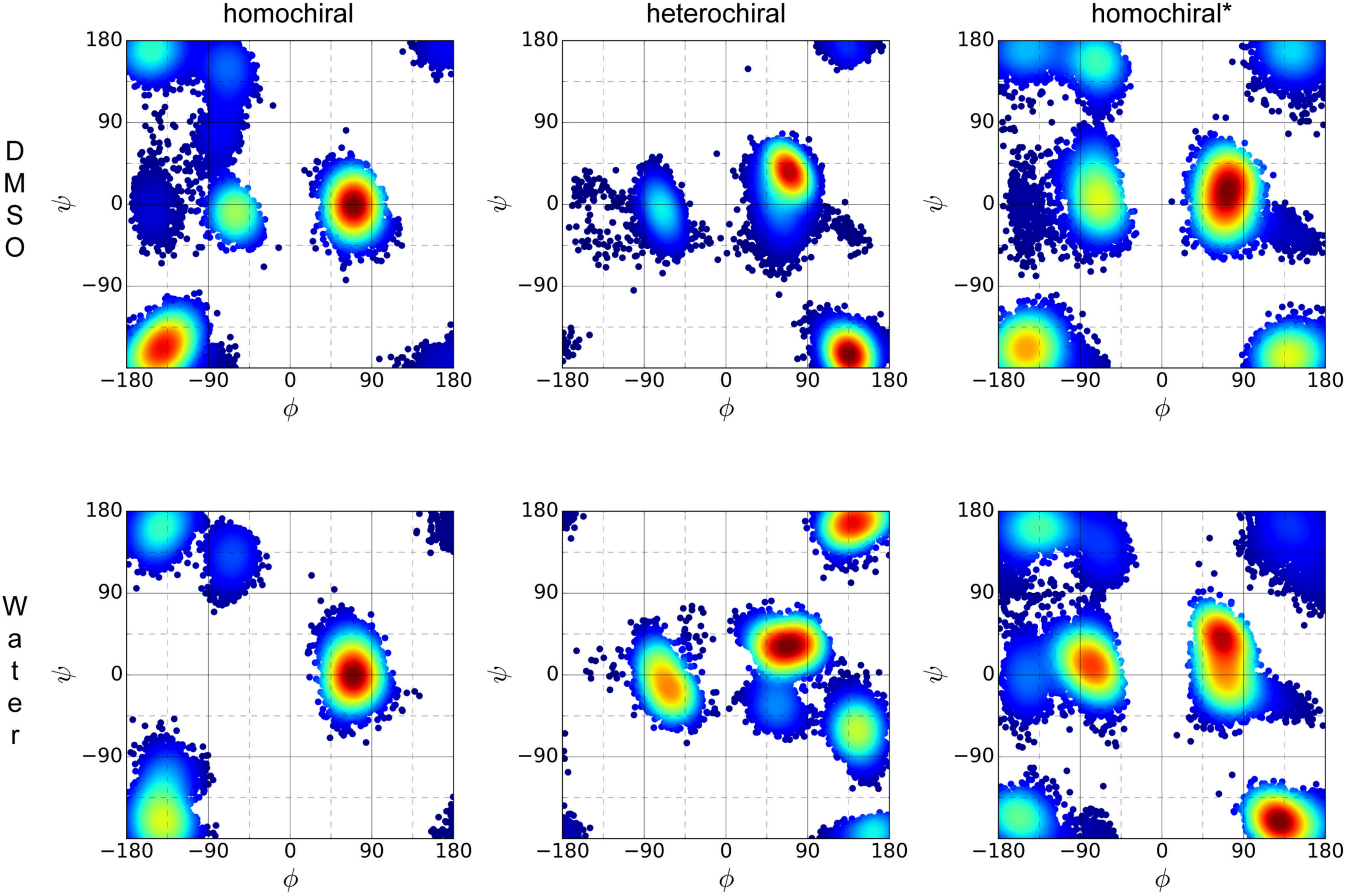
ϕ*/*ψ torsion angle distribution of the three representative molecules (homochiral: **10a**, heterochiral: **10b**, homochiral^*^: **10c**) for the last 100 ns of MD in DMSO (first row) and TIP4Pew water (second row).

The distribution of ϕ/ψ backbone torsional degrees of freedom is depicted in Figure 10 for each molecule in both solvents. Although the plots resemble Ramachandran distributions, a direct comparison with known patterns for standard peptides is not straightforward due to the introduction of the triazole moieties in the sequence. Nevertheless, the plots give a qualitative picture of the differences and similarities between the three variants considered in this analysis and of the effect of the solvent polarity on their conformational preferences. Overall, all three molecules appear to have their own unique signature in terms of distribution of backbone torsion angles. While distribution maxima depict distinct patterns for homo- and heterochiral, the glycine-containing derivative (homochiral^*^) shows features of both parent oligomers. The polarity of the solvent appears to have little to no effect on the ϕ/ψ distribution for all three molecules.

**Figure 11:**
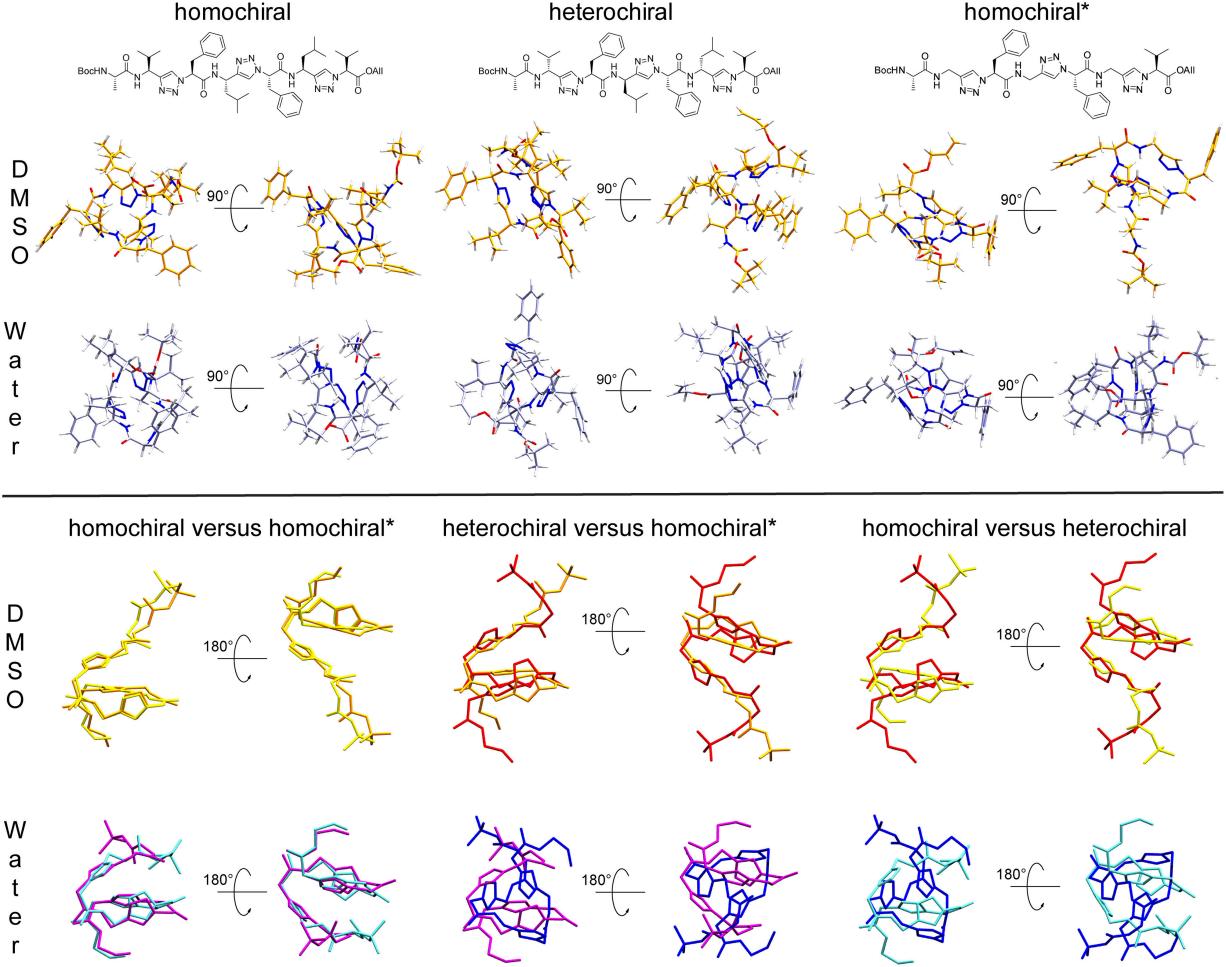
**Upper panel:** Representative structures for the main cluster of the three representative molecules (10a-c, homochiral, heterochiral, homochiral^*^) as obtained from backbone RMSD-based clustering over the last 100 ns of molecular dynamics in DMSO (orange) and TIP4Pew Water (iceblue). **Lower panel:** Alignment of the representative structure backbones for the homochiral and homochiral^*^, the heterochiral and homochiral^*^, as well as homo- and heterochiral oligomer. The homochiral oligomer is colored in yellow (DMSO) and cyan (Water), respectively, the heterochiral in red (DMSO) and blue (Water), respectively and the homochiral^*^ in orange (DMSO) and purple (Water), respectively.

Clustering based on the backbone RMSD of the oligomers for the second half of the simulations in DMSO and in water resulted in the following populations for the main structural clusters: 94 and 52% for the homochiral molecule in DMSO and in water, respectively, 71 and 59% for the heterochiral, and 60 and 76% for the homochiral^*^ compound. For the heterochiral compound in water, a second structural cluster was found with a population of 40% that differed from the main representative structure with a backbone RMSD value of 0.54 Å. The differences were localized on the flexible terminal residues and both structures can, therefore, be formally assigned to the same conformation with a total population of 99%, as confirmed by the rather flat RMSD and radius of gyration time-series (Supplementary Figures 10, 11). Similarly, the homochiral^*^ variant in DMSO results in a second main cluster with a trajectory fraction of 21% and a backbone RMSD value of 0.81 Å, primarily caused by both flexible ends pointing in orthogonal direction. Thus, the two main clusters can be joined with a combined trajectory fraction of 81%. Such large populations are consistent with the rigid character of the peptidomimetics already observed from the RMSD time series. To a given extent, the homochiral^*^ molecule appears to be slightly more flexible than the others when plunged in water. The representative structure for the main cluster of each simulation is depicted in the upper panel of Figure 11. In general, all oligomers considered in this analysis fold with a helically twisted conformation. Consistently with our observations thus far, the solvent has little to no effect on the conformation of the oligomers. The values of backbone torsional angles for the representative structure in DMSO and in water are listed in Table 1. The lower panel of Figure 11 shows a direct comparison of the backbone of the homochiral and homochiral^*^ variants, the heterochiral and homochiral^*^ ones, as well as a comparison between homo- and heterochiral ones. The figure illustrates that the homochiral and homochiral^*^ compounds fold into a nearly identical conformation in DMSO and in water, while the heterochiral molecule adopts a slightly different orientation of the triazole rings in DMSO and a significantly yet still compact conformation in water. Taking the homochiral molecule as reference, the homochiral^*^ and heterochiral compounds have a backbone RMSD value of 0.45 and 1.97 in DMSO, respectively, and of 1.27 and 3.61 Å in water.

**Table 1:** Dihedral angles of the representative structures for the main cluster of the three representative molecules (10a-c, homochiral, heterochiral, homochiral^*^) as obtained from molecular dynamics in DMSO and TIP4Pew Water.

	**Homochiral**		**Heterochiral**		Homochiral^*^	
	**Φ**	**Ψ**	**Φ**	**Ψ**	**Φ**	**Ψ**
**DMSO**						
Ala1	−61.9	132.4	−64.3	1.1	−78.8	158.2
V/G4n2	−148.7	−176.8	143.2	−169.5	−146.2	−170.3
F4c4	−58.6	−13.8	72.2	39.2	−58.2	2.8
L/G4n5	−117.6	−148.0	132.9	−163.6	−156.9	−142.0
F4c7	62.3	3.1	75.8	24.8	72.9	24.1
L/G4n8	−168.7	−171.1	142.8	−175.4	154.2	−166.1
V4c10	57.0	3.4	65.0	−13.3	62.1	0.0
**WATER**						
Ala1	−67.0	131.8	−97.4	19.5	−133.1	15.5
V/G4n2	−136.4	−112.8	167.0	−168.5	−130.4	165.5
F4c4	75.7	1.5	64.6	39.4	−67.1	22.5
L/G4n5	−155.0	175.3	131.4	−48.1	172.3	−153.7
F4c7	68.2	−3.8	71.6	37.5	65.0	37.4
L/G4n8	−152.0	−168.6	125.1	168.7	131.5	−153.5
V4c10	51.5	3.9	−51.1	−34.1	83.9	−15.2

**Figure 12:**
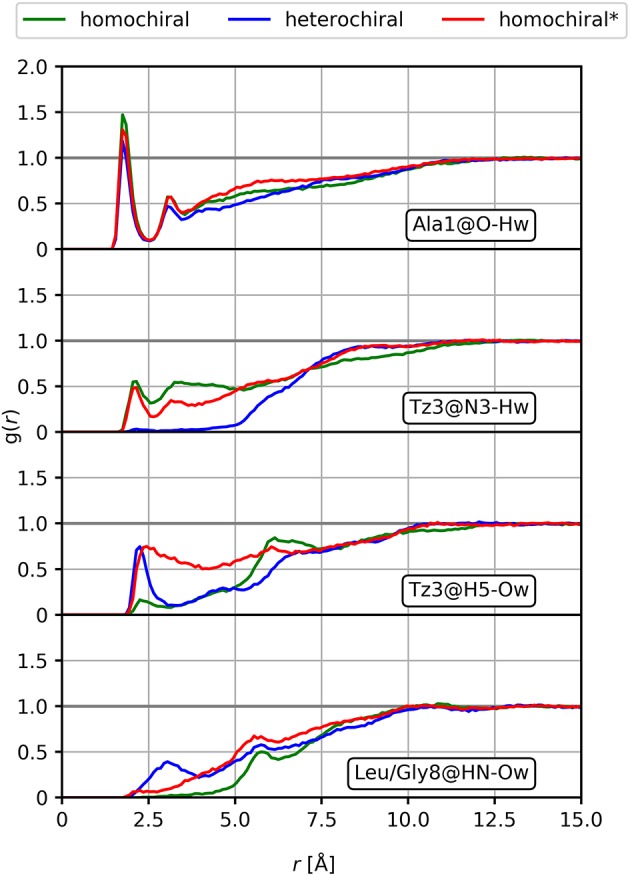
Radial distribution functions of selected interactions for the homochiral, the heterochiral, and the homochiral^*^ oligomers (10a-c, homochiral, heterochiral, homochiral^*^) in TIP4Pew water.

We further analyzed the potential solvation properties of the three oligomers in water by calculating the radial distribution functions (RDF) around key chemical groups. The complete results are available as supporting information (Supplementary Figures 12–14). In general, the RDFs show that the molecules are poorly solvated in water, which is expected regarding the highly hydrophobic character of the side chains. Only specific groups such as the backbone carbonyl moiety show stable interactions with one water molecule over the whole simulation with distances characteristic of hydrogen bonds. Such interactions are conserved over all three molecules, as illustrated by the RDF for the carbonyl group of the first alanine amino acid in Figure 12. Therein, we observe a sharp peak at about 1.8 Å, but no further structure formation at larger distances due to the hydrophobic character of the rest of the molecule. Among all the potential hydrogen bond donor/acceptor groups in the peptidomimetic considered in this work, we noted only few significant differences. (i) A shallow peak is present at short distances in the RDF of the nitrogen atom (N3) of the first triazole ring for both the homochiral and the homochiral^*^ molecules. The integration of this peak returns a value corresponding to a sporadic interaction with a water molecule occurring for about a third of the simulation time only. This weak interaction, however, is totally absent in the case of the heterochiral compound. (ii) Similar observations can be made regarding the hydrogen of that same triazole ring, which is involved in a weak interaction with water in two of the three molecules. As opposed to (i), however, this interaction is absent for the homochiral molecule and present for the two others. We also notice that, considering the sharpness of the peak, this interaction is tighter in the case of the heterochiral molecules than it is for the homochiral^*^ one. (iii) Comparison of the RDF around the amide nitrogen of the 8^th^ residue (i.e., Leu or Gly) indicates that this site is slightly more solvent accessible in the case of the heterochiral compound than it is for the two other molecules. These differences are due to a slightly better orientation of the corresponding chemical groups favoring interactions with the solvent and potentially with other polar compounds in different environments. Although these interactions with water are somewhat weak for these oligomers, the differences that we observed are indicative of potential interaction sites that could discriminate one molecule from another in a hypothetical protein environment.

Overall our conformational analysis revealed only little differences between the three variants of the oligomers under investigation. All three adopt a compact folded conformation in DMSO. The homochiral compound (**10a**) and the glycine containing analog (**10c**) adopt nearly identical conformations in DMSO, which tends to correlate with the observation of a similar pattern on the CD spectra for these two molecules. Considering the high hydrophobic character of the side chains, it is not surprising that the molecules remain folded in water in order to minimize the interaction surface area with the polar environment. This observation that the chirality of the different stereocenters has no significant impact on the conformation of the 1,4-disubstituted peptidotriazolamers is in contrast with our results for the 1,5-disubstituted counterpart (Kracker et al., 2018). There we identified that changes in the chirality of the α-carbons can induce drastic changes in the conformation of the molecules, i.e., while the homochiral hexapeptidotriazolamer Boc-AlaΨ[5Tz]Phe-ValΨ[5Tz]Ala-Leu-Ψ[5Tz]Val-OBzl formed a compact β-turn-like structure, the heterochiral equivalent was observed and predicted in an extended polyproline I-like conformation (Ke et al., 2012).

To further characterize the conformational properties of 1,4-disubstituted peptidotriazolamers, we analyzed a longer, hypothetical oligomer bearing only methyl side chains to mimic a poly-alanine peptide Figure 8). Similarly, to the case of shorter molecules, we considered three variants labeled as homochiral, heterochiral, and homochiral^*^. Due to the significantly larger conformational space of these oligomers, we performed four parallel 200 ns simulations for each molecule in each of the two solvents (i.e., in DMSO and in water, for a total of 24 simulations) in order to reach a statistically significant sampling.

**Figure 13:**
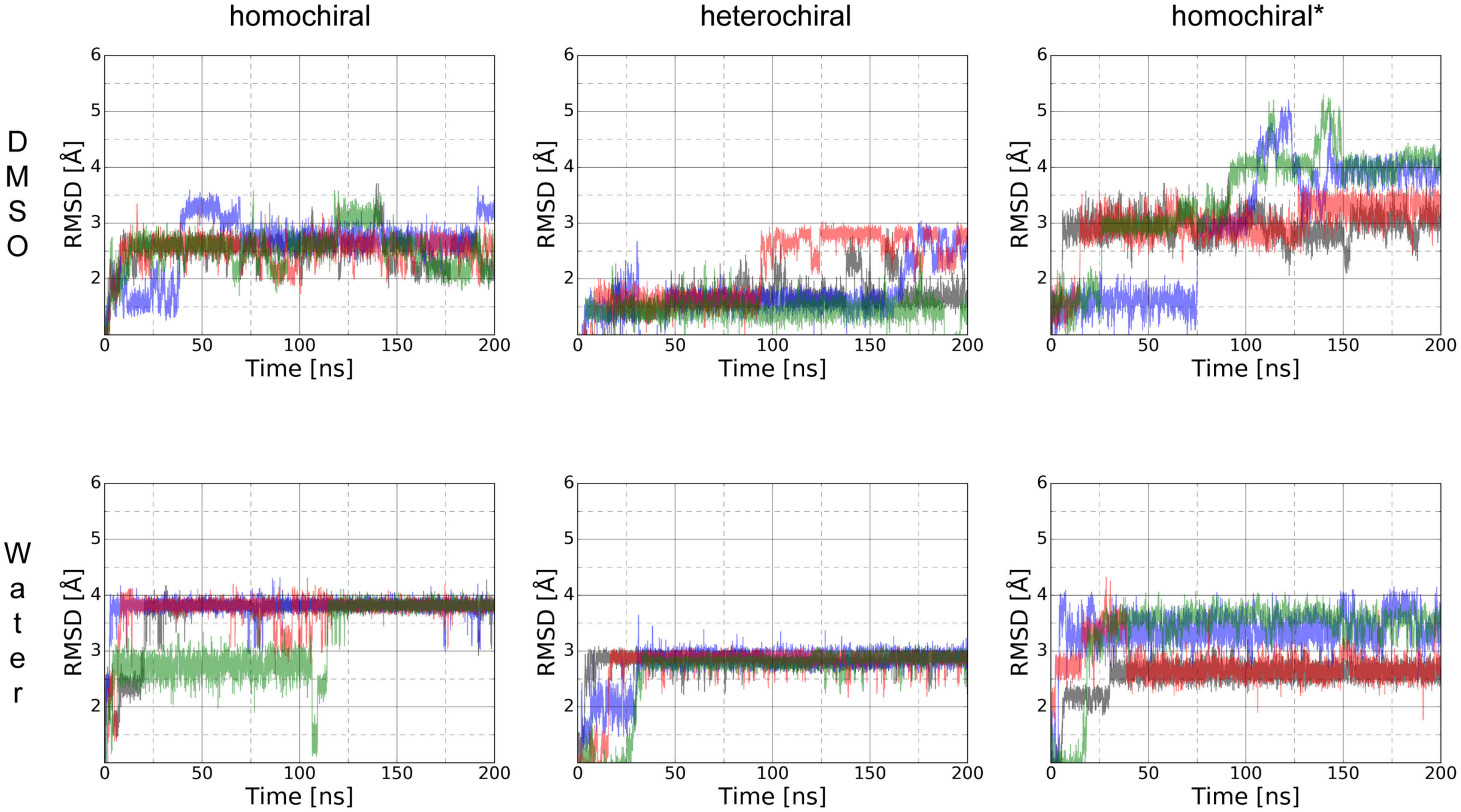
RMSD time series for the whole trajectory of homochiral, heterochiral, and homochiral^*^ poly-alanine derivatives in DMSO (first row) and TIP4Pew water (second row) for Poly-Ala's homochiral, heterochiral and homochiral^*^ equivalent. The starting point of the trajectory is taken as reference point.

The RMSD time series for the whole trajectory of each replicate simulation are shown in Figure 13. As expected, the simulations took longer to reach their local equilibrium compared to the case of the smaller molecules. After 150 ns, all trajectories reach a stable value of RMSD or oscillate between two conformational basins in a balanced manner. Compared to the case of shorter oligomers in which we had NMR information to produce relevant initial structures, the lack of experimental data in the present case translates into higher RMSD values since our initial geometries are likely to be far from a minimum conformation. Nevertheless, all simulations reached equilibrium and yield a distribution of stable conformers.

In contrast to the case of short oligomers, the homo- and heterochiral polyalanine derivatives suffer from a significant decrease of flexibility when moving from DMSO to water. This is illustrated in Figure 13 by the fact that all four simulations systematically converge toward the same value of RMSD in water only. Once this stable plateau is reached, the amplitude of fluctuations also decreases, showing the tight conformational restraints exerted by the solvent on the solute. Such a drastic effect of the solvent was, however, not observed in the case of the glycine-containing variant (homochiral^*^), which is most likely due to the significantly reduced hydrophobic character of that molecule. ROG time series are available as supporting information (Supplementary Figure 15) for the whole trajectories and show that even significant fluctuations in RMSD do not result in large changes in the compactness of the conformations. The ϕ*/*ψ torsion angle distributions (Supplementary Figure 16) are in a good agreement with the RMSD fluctuations, showing that all three molecules have a unique conformational signature.

We performed the clustering analysis over the last 50 ns of the trajectories of all four replica. The main structural clusters in DMSO and water showed a population of 55 and 95%, respectively for the homochiral, 72 and 91% for the heterochiral, and 52 and 40% for the homochiral^*^ oligomer. For the homochiral^*^ molecule in water three other clusters with significant population (i.e., 32 and 27%) were identified, but showing a high similarity with the main cluster as indicated by backbone RMSD values as low as 1.4 and 0.8 Å.

**Figure 14:**
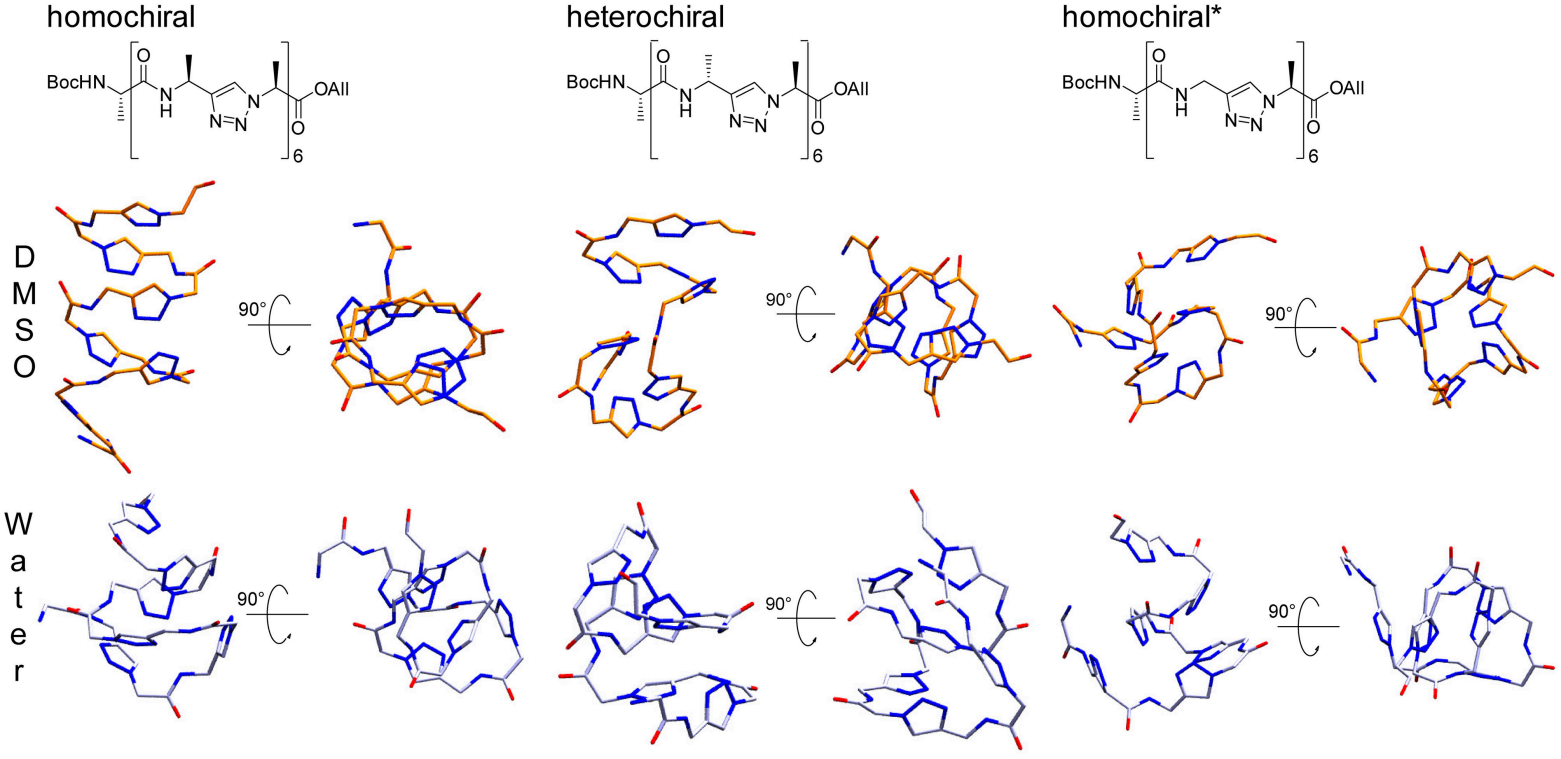
Representative structures for the main cluster of the three representative polyalanine derivatives, as obtained from molecular dynamics in DMSO (orange) and TIP4Pew Water (iceblue). The clustering was performed over the last 50 ns, based on the backbone atoms (C^O^, N, C^α^, N^1^, N^2^, N^3^) only.

The representative structures are shown in Figure 14. The homochiral molecule shows a clear helical conformation in DMSO, which collapses in water. The heterochiral molecule shows a compactness in DMSO similar to that of the homochiral one. However, the conformation of the former describes a twisted “S”-shape, which also collapses in water. The Gly-substituted oligomer shows no clear secondary structure in either of the solvents, which is in agreement with the more flexible character of this molecule as interpreted from the RMSD time series.

## Conclusion

Herein we present a new class of peptidomimetics containing 1,4-disubstituted 1*H*-1,2,3-triazoles and amide bonds in an alternating fashion. The synthesis is based on chiral propargylamines and chiral α-azido acids. Beside the introduction of a versatile and robust synthetic strategy we elucidated their foldamer properties by means of molecular dynamics simulations in explicit solvent. Homo- and heterochiral tetra-, hexa-, and heptamers based on two different sequences as well as a homochiral oligomer, in which every second sidechain is replaced by a proton, have been synthesized based on a submonomer approach. Molecular modeling of the Boc-Ala-ValΨ[4Tz]Phe-LeuΨ[4Tz]Phe-LeuΨ[4Tz]Val-OAll sequence (homo- and heterochiral) as well as a Gly-substituted derivative revealed a compact folded conformation in DMSO, similar for all three variants. The conformation of these molecules remains compactly folded in water as well, due to the hydrophobic character of the non-polar side chains. In addition, we predicted the conformations of homo-, heterochiral, and glycine-containing poly-alanine peptidotriazole oligomers. In contrast to the shorter oligomers, our calculations suggest a well-defined and significantly different secondary structure for the homo- (helix) and heterochiral (twisted S) variants in DMSO, which collapses in water. The glycine-substituted oligomers, however, shows no clear secondary structure and a significantly higher flexibility compared to the two other variants, with a conformational space that is little-affected by the solvent's polarity.

## Experimental Section

### Synthesis

All chemicals were purchased from *Sigma Aldrich* (Taufkirchen, Germany), *Acros* (Geel, Belgium), *Alfa Aesar* (Ward Hill, USA), *VWR* (Darmstadt, Germany), *Chempur* (Karlsruhe, Germany), *Iris Biotech* (Marktredwitz, Germany), *Bachem* (Bubendorf, Switzerland), and were employed without additional purification. Moisture- and air-sensitive reaction steps were conducted in flame-dried glassware and under argon atmosphere. Dichloromethane and toluene were freshly distilled from CaH_2_ and Na, respectively. THF was pre-dried over KOH followed by drying over sodium/benzophenone under reflux conditions and was distilled freshly prior usage.

Analytical RP-HPLC was performed on a *Thermo Separation Products* system equipped with a UV-6000 LP detector, a P-4000 pump, a Hypersil Gold 3 μm (C18; 150 × 2.1 mm) column. A flow rate of 0.7 mL min^−1^ using Eluent A: H_2_O/CH_3_CN/TFA (95/5/0.1) and Eluent B: CH_3_CN/H_2_O/TFA (95/5/0.1) was employed. Method A1: 0–3 min (100% 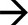 A 0% A), 3–4 min (0% A), 4–5 min (0% A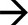 100% A). Method A2: 0–0.5 min (100% A), 0.5–7.5 min (100% A 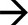 0% A), 7.5–8.5 min (0% A), 8.5–9.5 min (0% A 100%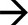 A), 9.5–10 min (100% A).

Preparative RP-HPLC was performed on a *Hitachi MERCK LaChrom* system equipped with a UV-Vis L-7420 detector, a L-7150 pump and a *Phenomenex* Jupiter 10 μm column (C18; 300 Å, 250 × 21.1 mm). A flow rate of 10.0 mL min^−1^ using Eluent A: H_2_O/CH_3_CN/TFA (95/5/0.1) and Eluent B: CH_3_CN/H_2_O/TFA (95/5/0.1) was employed. Method P1: 0–3 min (100% A 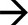 75% A), 3–20 min (75% A 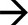 0% A), 20–100 min (0% A), 100–110 min (0% A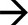 100% A). Method P2: 0–3 min (100% A), 3–35 min (100% A 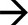 15% A), 35–100 min (15% A), 35–100 min (15% A), 100–110 min (15% A 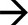 100% A). Method P3: 0–5 min (100% A), 5–35 min (100% A 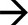 0% A), 35–40 min (0% A), 40–45 min (0% A 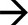 100% A).

In addition, a *Thermo Separation Products* system equipped with a UV-1000 detector, a P-4000 pump, was used utilizing the same methods.

NMR spectra were recorded at 298 K on a DRX 500 (^1^H: 500 MHz, ^13^C: 126 MHz), an Avance III 500 (^1^H: 500 MHz, ^13^C: 126 MHz), and an Avance 600 spectrometer (^1^H: 600 MHz, ^13^C: 151 MHz) (*Bruker Biospin*, Rheinstetten, Germany). Chemical shifts are reported relative to residual solvent peaks (DMSO-d6: ^1^H: 2.50 ppm, ^13^C: 39.52 ppm; CDCl_3_: ^1^H: 7.26 ppm, ^13^C: 77.16 ppm) (Gottlieb et al., 1997). ^1^H- and ^13^C-NMR spectra (Supplementary Figures 1–9) as well as ^1^H-^1^H-COSY-, ^1^H-^1^H-TOCSY, ^1^H-^13^C-HMBC, and ^1^H-^13^C-HMQC-NMR spectra were used for the signal assignments. ^1^H-^1^H-ROESY-NMR spectra were only taken for the restraints list.

ESI/APCI mass spectra were recorded using an Esquire 3000 ion trap mass spectrometer (*Bruker Daltonik GmbH*, Bremen, Germany) equipped with a standard ESI/APCI source. Samples were introduced directly with a syringe pump. Nitrogen served both as the nebulizer gas and the dry gas. Nitrogen was generated using a Bruker nitrogen generator NGM 11. Helium served as cooling gas for the ion trap and collision gas for MS experiments.

MALDI TOF mass spectra were recorded with a Ultraflex MALDI-TOF/TOF mass spectrometer (*Bruker Daltonik GmbH*, Bremen, Germany) operated in reflectron mode. 2,5-Dihydroxybenzoic acid was used as the matrix. Ionization was achieved using a LTB nitrogen laser MNL 200 (337 nm beam wavelength, 50 Hz repetition rate).

High-resolution masses were recorded using a MicroToF mass spectrometer (*Bruker Daltonik GmbH*, Bremen, Germany) with sample-loop injection. Mass calibration was performed immediately prior measurement with sodium formate cluster and quasi-internal calibration.

CD-spectra were recorded on a *JASCO* J-810 spectrometer in acetonitrile (HPLC grade) at 20°C.

#### General Procedures

Bus-protected propargylamines were synthesized according to Wünsch et al. (2017) as well as the α-azido acids in the style of Kracker et al. (2018).

**GP-1:** The azide (1.1 equivalent) and the alkyne (1.0 equivalent) were dissolved in DMF/H_2_0 (10:1, 0.1 M solution). Afterwards, CuSO_4_·5H_2_O (0.5 equivalents) and sodium ascorbate (1.0 equivalent) are added. The mixture was stirred 15 h at room temperature. After completion of the reaction the solvent was evaporated. The residue was dissolved with saturated NaCl-solution. After extraction with DCM (3×), drying over Na_2_SO_4_, and evaporation of the solvent, the pure triazole was delivered after purification via preparative HPLC or column chromatography.

**GP-2:** The free amine was dissolved in DMF (0.1 M solution). After addition of HATU (1.5 equivalents), HOAt (1.5 equivalents), DIPEA (9.0 equivalents) and an azidocarboxylic acid (1.1 equivalents) the reaction mixture is stirred 15 h at rt. After evaporation of the solvent purification via preparative HPLC or column chromatography yields the pure amide.

#### Explicit Experimental Procedures

**(*S*)-Bus-LeuΨ[4Tz]Val-OAll (3a):** N_2_ = Val-OAll (1a, 132 mg, 0.720 mmol), (*S*)-Bus-Leu-CH (2a, 104 mg, 0.483 mmol), CuSO_4_·5H_2_O (60 mg, 0.24 mmol) and sodium ascorbate (96 mg, 0.48 mmol) were stirred according to **GP-1**. Purification via column chromatography (DCM/MeOH 80:1, *R*_*f*_ = 0.13) yielded the product as yellow oil. Yield: 180 mg (0.452 mmol, 94%); ^1^H-NMR (500 MHz, CDCl_3_): δ [ppm] = 7.88 (s, 1H); 5.88 (m, 1H); 5.31 (dddd, ^4^*J* = 1.3 Hz, 1.3 Hz, ^3^*J* = 17.1 Hz, ^2^*J* = 1.1 Hz, 1H); 5.25 (dddd, ^4^*J* = 1.3 Hz, 1.3 Hz, ^3^*J* = 10.5 Hz, ^2^*J* = 1.1 Hz, 1H); 5.10 (d, ^3^*J* = 8.8 Hz, 1H); 4.68–4.60 (m, 2H); 4.53 (m, 1H); 3.69 (d, ^3^*J* = 8.1 Hz, 1H); 2.47 (m, 1H); 1.90–1.77 (m, 2H); 1.68 (m, 1H); 1.19 (s, 9H); 1.00 (d, ^3^*J* = 6.7 Hz, 3H); 0.91 (d, ^3^*J* = 6.7 Hz, 3H); 0.89 (d, ^3^*J* = 6.6 Hz, 3H); 0.83 (d, ^3^*J* = 6.8 Hz, 3H); ^13^C-NMR (126 MHz, CDCl_3_): δ [ppm] = 168.5; 150.2; 131.0; 121.3; 119.8; 68.9; 66.6; 56.4; 51.4; 45.5; 32.5; 24.6; 22.9; 22.8; 21.9; 19.3; 18.8; MS (ESI): *m/z* = 421.3 ([M+Na]^+^); HRMS (ESI): *m/z* calcd. for C_19_H_34_N_4_O_3_SNa^+^ ([M+Na]^+^): 421.2244, found: 421.2253.

**N**_**2**_**=Phe-LeuΨ[4Tz]Val-OAll (6a):** (*S*)-Bus-LeuΨ[4Tz]Val-OAll (3a, 171 mg, 0.429 mmol) was dissolved in MeOH (4 mL). After addition of 4 M HCl in dioxane (2.15 mL, 8.6 mmol) the mixture was stirred for 2 h at rt. After evaporation of the solvent the product was used for the next step without further purification. ^1^H-NMR (500 MHz, CDCl_3_): δ [ppm] = 8.53 (s, 3H); 8.09 (s, 1H); 5.89 (m, 1H); 5.33 (dddd, ^4^*J* = 1.4 Hz, 1.4 Hz, ^3^*J* = 17.2 Hz, ^2^*J* = 1.2 Hz, 1H); 5.27 (dddd, ^4^*J* = 1.2 Hz, 1.2 Hz, ^3^*J* = 10.3 Hz, ^2^*J* = 1.0 Hz, 1H); 5.14 (d, ^3^*J* = 8.5 Hz, 1H); 4.71–4.60 (m, 3H); 2.49 (m, 1H); 2.00 (m, 1H); 1.90 (m, 1H); 1.48 (m, 1H); 0.99 (d, ^3^*J* = 6.7 Hz, 3H); 0.90 (d, ^3^*J* = 6.5 Hz, 3H); 0.87 (d, ^3^*J* = 6.6 Hz, 3H); 0.81 (d, ^3^*J* = 6.7 Hz, 3H).

H-LeuΨ[4Tz]Val-OAll·HCl was stirred with the azido carboxylic acid N_2_ = Phe-OH (1b, 90 mg, 0.47 mmol), HATU (247 mg, 0.650 mmol), HOAt (88 mg, 0.65 mmol) and DIPEA (0.65 mL, 3.87 mmol) according to **GP-2**. Purification via column chromatography (DCM/MeOH 50:1, *R*_*f*_ = 0.27) yielded the product as colorless solid. Yield: 181 mg (0.387 mmol, 86%); ^1^H-NMR (500 MHz, CDCl_3_): δ [ppm] = 7.75 (s, 1H); 7.32–7.25 (m, 5H); 6.80 (d, ^3^*J* = 8.7 Hz, 1H); 5.89 (m, 1H); 5.33 (dddd, ^4^*J* = 1.5 Hz, 1.5 Hz, ^3^*J* = 17.1 Hz, ^2^*J* = 1.1 Hz, 1H); 5.28 (dddd, ^4^*J* = 1.5 Hz, 1.5 Hz, ^3^*J* = 10.2 Hz, ^2^*J* = 1.0 Hz, 1H); 5.21 (m, 1H); 5.13 (d, ^3^*J* = 8.7 Hz, 1H); 4.71–4.62 (m, 2H); 4.18 (dd, ^3^*J* = 7.8 Hz, 4.2 Hz, 1H); 3.32 (dd, ^3^*J* = 4.3 Hz, ^2^*J* = 14.0 Hz, 1H); 3.06 (dd, ^3^*J* = 8.1 Hz, ^2^*J* = 14.2 Hz, 1H); 2.47 (m, 1H); 1.70–1.67 (m, 2H); 1.34 (m, 1H); 1.02 (d, ^3^*J* = 6.7 Hz, 3H), 0.89 (d, ^3^*J* = 6.6 Hz, 3H); 0.88 (d, ^3^*J* = 6.5 Hz, 3H); 0.82 (d, ^3^*J* = 6.7 Hz, 3H); ^13^C-NMR (126 MHz, CDCl_3_): δ [ppm] = 168.5; 167.7; 148.2; 136.1; 131.0; 129.7; 128.7; 127.3; 120.8; 119.9; 68.9; 66.6; 65.5; 44.1; 44.0; 38.5; 32.5; 24.7; 22.7; 22.3; 19.3; 18.8; MS (ESI): *m/z* = 490.2 ([M+Na]^+^'); HRMS (ESI): *m/z* calcd. for C_24_H_33_N_7_O_3_Na^+^ ([M+Na]^+^): 490.2537, found: 490.2542.

**(*S*)-Bus-Leuψ[4Tz]Phe-LeuΨ[4Tz]Val-OAll (7a):** N_2_ = Phe-LeuΨ[4Tz]Val-OAll (6a, 181 mg, 0.387 mmol) was stirred with the alkyne (*S*)-Bus-Leu-CH (2a, 92 mg, 0.43 mmol), CuSO_4_·5H_2_O (50 mg, 0.20 mmol) and sodium ascorbate (77 mg, 0.39 mmol) according to **GP-1**. Purification via preparative RP-HPLC yielded the product as colorless solid. Yield: 155 mg (0.227 mmol, 59%); Analytical RP-HPLC(Method A1): *t*_*R*_ = 3.49 min; Preparative RP-HPLC(Method P2, 220 nm): *t*_*R*_ = 42.0 min; ^1^H-NMR (500 MHz, CDCl_3_): δ [ppm] = 7.67 (s, 1H); 7.59 (s, 1H); 7.15–7.14 (m, 3H); 7.06 (d, ^3^*J* = 8.4 Hz, 1H); 6.99–6.97 (m, 2H); 5.90 (m, 1H); 5.32 (dddd, ^4^*J* = 1.2 Hz, 1.2 Hz, ^3^*J* = 17.1 Hz, ^2^*J* = 1.6 Hz, 1H); 5.28 (dddd, ^4^*J* = 1.2 Hz, 1.2 Hz, ^3^*J* = 10.3 Hz, ^2^*J* = 1.2 Hz, 1H); 5.26–5.19 (m, 2H) 5.10 (d, ^3^*J* = 8.8 Hz, l1H); 4.71–4.61 (m, 2H); 4.46 (m, 1H); 3.88 (m, 1H); 3.56 (dd, ^3^*J* = 6.5 Hz, ^2^*J* = 14.1 Hz, 1H); 3.38 (dd, ^3^*J* = 9.2 Hz, ^2^*J* = 14.1 Hz, 1H); 2.47 (m, 1H); 1.86–1.77 (m, 2H); 1.74–1.70 (m, 2H); 1.58 (m, 1H); 1.45 (m, 1H); 1.19 (s, 9H); 1.01 (d, ^3^*J* = 6.7 Hz, 3H), 0.91 (m, 12 H); 0.81 (d, ^3^*J* = 6.7 Hz, 3H); ^13^C-NMR (126 MHz, CDCl_3_): δ [ppm] = 168.4; 166.8; 149.7; 148.2; 135.7; 131.0; 129.1; 128.8; 127.3; 122.7; 121.1; 119.9; 69.0; 66.7; 66.1; 56.5; 51.4; 45.1; 44.6; 43.8; 39.1; 32.5; 24.9; 24.6; 22.8; 22.7; 22.7; 22.2; 21.2; 19.3; 18.8; MS (ESI): *m/z* = 705.4 ([M+Na]^+^); HRMS(ESI): *m/z* calcd. for C_35_H_54_N_8_O_4_SNa^+^ ([M+Na]^+^): 705.3881, found: 705.3871.

**N**_**2**_**=Phe-LeuΨ[4Tz]Phe-LeuΨ[4Tz]Val-OAll (8a):** (*S*)-Bus-LeuΨ[4Tz]Phe-LeuΨ[4Tz]Val-OAll (7a, 33 mg, 0.048 mmol) was dissolved in MeOH (2 mL) and stirred at rt with 4 M HCl in dioxane (24 μL solution). After 2 h the solvent was evaporated. The free amine was dissolved in DMF and stirred with the azido carboxylic N_2_ = Phe-OH (1b, 10 mg, 0.052 mmol), HATU (28 mg, 0.073 mmol), HOAt (10 mg, 0.073 mmol) and DIPEA (74 μL, 0.44 mmol) according to **GP-2**. Purification via preparative RP-HPLC yielded the product as colorless solid. Yield: 10 mg (13 μmol, 27%); Preparative RP-HPLC: *t*_*R*_ = 27.2 min (Method P1, 220 nm); ^1^H-NMR (500 MHz, DMSO-*d*_6_): δ [ppm] = 8.94 (d, ^3^*J* = 8.4 Hz, 1H); 8.56 (d, ^3^*J* = 8.6 Hz, 1H); 7.98 (s, 1H); 7.93 (s, 1H); 7.29–7.26 (m, 2H); 7.22–7.21 (m, 3H); 7.17–7.11 (m, 5H); 5.90 (m, 1H); 5.61 (dd, ^3^*J* = 9.5 Hz, 6.4 Hz, 1H); 5.29–5.20 (m, 3H); 5.06–5.01 (m, 2H); 4.70–4.63 (m, 2H); 4.00 (dd, ^3^*J* = 9.0 Hz, 5.5 Hz, 1H); 3.35 (dd, ^3^*J* = 6.4 Hz, ^2^*J* = 14.2 Hz, 1H); 3.30 (dd, ^3^*J* = 9.4 Hz, ^2^*J* = 14.0 Hz, 1H); 3.02 (dd, ^3^*J* = 5.4 Hz, ^2^*J* = 13.9 Hz, 1H); 2.90 (dd, ^3^*J* = 9.0 Hz, ^2^*J* = 13.9 Hz, 1H); 2.57 (m, 1H); 1.73–1.60 (m, 4H); 1.47–1.37 (m, 2H); 0.96 (d, ^3^*J* = 6.7 Hz, 3H), 0.88 (m, 6H); 0.83 (d, ^3^*J* = 6.6 Hz, 3H); 0.72 (m, 6H); ^13^C-NMR (126 MHz, DMSO-*d*_6_): δ [ppm] = 168.2; 167.9; 166.7; 148.1; 147.9; 136.8; 136.1; 133.8; 129.0; 128.9; 128.4; 128.2; 126.7; 126.7; 122.1; 121.3; 118.4; 67.6; 65.7; 63.7; 62.1; 43.7; 43.6; 43.4; 43.4; 37.4; 36.9; 30.6; 24.3; 24.2; 22.6; 22.6; 21.9; 21.8; 18.8; 18.2; MS (ESI): *m/z* = 774.4 ([M+Na]^+^).

**(*S*)-Bus-ValΨ[4Tz]Phe-LeuΨ*[4Tz]*Phe-LeuΨ[4Tz]Val-OAll (9a):** N_2_ = Phe-LeuΨ[4Tz]Phe-LeuΨ[4Tz]Val-OAll (8a, 10 mg, 13 μmol) was stirred with the (*S*)-Bus-Val-CH (2b, 4.0 mg, 20 μmol), CuSO_4_·5H_2_O (2.5 mg, 10 μmol) and sodium ascorbate (2.6 mg, 13 μmol) according to **GP-1**. Purification via preparative RP-HPLC yielded the product as colorless solid. Yield: 6.0 mg (6.3 μmol, 48%); Analytical RP-HPLC: *t*_*R*_ = 3.60 min (Method A1); Preparative RP-HPLC: *t*_*R*_ = 43.4 min (Method P2, 220 nm); ^1^H-NMR (600 MHz, DMSO-*d*_6_): δ [ppm] = 8.94 (d, ^3^*J* = 8.5 Hz, 1H); 8.83 (d, ^3^*J* = 8.5 Hz, 1H); 8.15 (s, 1H); 8.00 (s, 1H); 7.93 (s, 1H); 7.18–7.09 (m, 10H); 5.90 (m, 1H); 5.59 (dd, ^3^*J* = 9.5 Hz, 6.2 Hz, 1H); 5.55 (dd, ^3^*J* = 9.5 Hz, 6.3 Hz, 1H); 5.28 (d, ^3^*J* = 8.5 Hz, 1H); 5.27 (dddd, ^4^*J* = 1.5 Hz, 1.5 Hz, ^3^*J* = 17.2 Hz, ^2^*J* = 1.5 Hz, 1H); 5.23 (s *(br)*, 1H); 5.21 (dddd, ^4^*J* = 1.3 Hz, 1.3 Hz, ^3^*J* = 10.5 Hz, ^2^*J* = 1.4 Hz, 1H); 5.06–4.99 (m, 2H); 4.70–4.64 (m, 2H); 4.12 (dd, ^3^*J* = 7.0 Hz, 7.0 Hz, 1H); 3.37–3.24 (m, 4H); 2.57 (m, 1H); 2.07 (m, 1H); 1.70 (m, 1H); 1.67–1.62 (m, 2H); 1.59 (m, 1H); 1.43 (m, 1H); 1.33 (m, 1H); 1.11 (s, 9H); 0.96 (d, ^3^*J* = 6.7 Hz, 3H), 0.84 (d, ^3^*J* = 6.7 Hz, 3H); 0.83–0.78 (m, 15H); 0.74 (d, ^3^*J* = 6.7 Hz, 3H); ^13^C-NMR (151 MHz, DMSO-*d*_6_): δ [ppm] = 167.8; 166.7; 166.6; 148.3; 148.1; 147.8; 136.1; 136.1; 131.8; 128.9; 128.9; 128.2; 128.2; 126.7; 126.7; 122.0; 122.0; 121.2; 118.4; 67.5; 65.7; 63.7; 63.7; 57.9; 55.7; 43.7; 43.7; 43.1; 43.4; 37.8; 37.4; 33.4; 30.6; 24.3; 24.1; 22.7; 22.5; 22.4; 21.9; 21.8; 18.8; 18.8; 18.6; 18.3; MS (ESI): *m/z* = 975.5 ([M+Na]^+^).

**Boc-Ala-ValΨ[4Tz]Phe-LeuΨ[4Tz]Phe-LeuΨ[4Tz]Val-OAll (10a):** *(S*)-Bus-ValΨ[4Tz]Phe-LeuΨ[4Tz]Phe-LeuΨ[4Tz]Val-OAll (9a, 5.0 mg, 5.2 μmol) was dissolved in MeOH (0.5 mL) and 4 M HCl in dioxane (26 μL solution) was added. After 2 h stirring at rt the solvent was evaporated and the free amine was dissolved in DMF. It was stirred with Boc-Ala-OH (1.5 mg, 7.9 μmol), HATU (3.0 mg, 7.9 μmol), HOAt (1.0 mg, 7.3 μmol) and DIPEA (8.0 μL, 47 μmol) according to **GP-2**. Purification via preparative RP-HPLC yielded the product as colorless solid. Yield: 3.0 mg (2.9 μmol, 57%); Preparative RP-HPLC: *t*_*R*_ = 42.5 min (Method P2, 220 nm); ^1^H-NMR (600 MHz, DMSO-*d*_6_): δ [ppm] = 8.93 (d, ^3^*J* = 8.4 Hz, 1H); 8.87 (d, ^3^*J* = 8.5 Hz, 1H); 8.05 (s, 1H); 8.01 (s, 1H); 7.93 (s, 1H); 7.93 (d, ^3^*J* = 9.1 Hz, 1H); 7.18–7.08 (m, 10H); 6.93 (d, ^3^*J* = 7.7 Hz, 1H); 5.90 (m, 1H); 5.59 (dd, ^3^*J* = 9.5 Hz, 6.2 Hz, 1H); 5.56 (dd, ^3^*J* = 9.9 Hz, 5.8 Hz, 1H); 5.28 (d, ^3^*J* = 8.4 Hz, 1H); 5.27 (dddd, ^4^*J* = 1.5 Hz, 1.5 Hz, ^3^*J* = 17.2 Hz, ^2^*J* = 1.5 Hz, 1H); 5.22 (dddd, ^4^*J* = 1.2 Hz, 1.2 Hz, ^3^*J* = 10.4 Hz, ^2^*J* = 1.6 Hz, 1H); 5.04 (m, 1H); 5.00 (m, 1H); 4.81 (dd, ^3^*J* = 9.1 Hz, 7.0 Hz, 1H); 4.70–4.64 (m, 2H); 4.02 (m, 1H); 3.37–3.22 (m, 4H); 2.57 (m, 1H); 2.00 (m, 1H); 1.71 (m, 1H); 1.67–1.62 (m, 2H); 1.57 (m, 1H); 1.42 (m, 1H); 1.37 (s, 9H); 1.32 (m, 1H); 1.14 (d, ^3^*J* = 7.1 Hz, 3H); 0.96 (d, ^3^*J* = 6.7 Hz, 3H); 0.84 (d, ^3^*J* = 6.7 Hz, 3H); 0.81–0.76 (m, 15H); 0.73 (d, ^3^*J* = 6.7 Hz, 3H); ^13^C-NMR (151 MHz, DMSO-*d*_6_): δ [ppm] = 172.5; 168.2; 167.2; 167.1; 154.4; 148.5; 148.2; 147.5; 136.5; 136.2; 132.4; 130.1; 129.3; 129.0; 128.6; 127.6; 127.3; 122.4; 121.8; 121.7; 118.9; 78.4; 68.1; 66.1; 64.2; 64.2; 51.2; 50.2; 44.1; 44.1; 43.9; 43.8; 38.5; 37.9; 33.1; 31.1; 29.0; 25.0; 24.4; 23.0; 23.0; 22.2; 22.2; 19.2; 18.7; 18.5; 18.4; 18.0; MS(ESI): *m/z* = 1042.6 ([M+Na]^+^); HRMS(ESI): *m/z* calcd. for C_54_H_77_N_13_O_7_Na^+^ ([M+Na]^+^): 1042.5967, found: 1042.5967.

**(*R*)-Bus-****d****-LeuΨ[4Tz]Val-OAll (3b):** The azide N_2_ = Val-OAll (1a, 70 mg, 0.38 mmol) was stirred with the alkyne (*R*)-Bus-D-Leu-CH (2c, 41 mg, 0.19 mmol), CuSO_4_·5H_2_O (24 mg, 96 μmol) and sodium ascorbate (38 mg, 0.19 mmol) according to **GP-1**. Purification via column chromatography (DCM/MeOH 80:1, *R*_*f*_ = 0.18) yielded the product as yellow oil. Yield: 74 mg (0.19 mmol, 98%); ^1^H-NMR (500 MHz, CDCl_3_): δ [ppm] = 7.90 (s, 1H); 5.84 (m, 1H); 5.26 (dddd, ^4^*J* = 1.4 Hz, 1.4 Hz, ^3^*J* = 17.3 Hz, ^2^*J* = 1.3 Hz, 1H); 5.20 (dddd, ^4^*J* = 1.2 Hz, 1.2 Hz, ^3^*J* = 10.4 Hz, ^2^*J* = 1.1 Hz, 1H); 5.06 (d, ^3^*J* = 8.6 Hz, 1H); 4.74–4.64 (m, 2H); 4.55 (m, 1H); 3.61 (d, ^3^*J* = 6.8 Hz, 1H); 2.43 (m, 1H); 1.85 (m, 1H); 1.77 (m, 1H); 1.70 (m, 1H); 1.19 (s, 9H); 0.94 (d, ^3^*J* = 6.7 Hz, 3H); 0.91 (d, ^3^*J* = 6.5 Hz, 3H); 0.90 (d, ^3^*J* = 6.5 Hz, 3H); 0.78 (d, ^3^*J* = 6.7 Hz, 3H); ^13^C-NMR (126 MHz, CDCl_3_): δ [ppm] = 168.4; 150.7; 131.0; 121.6; 119.7; 68.8; 66.5; 56.4; 51.7; 45.7; 32.4; 24.5; 23.0; 22.7; 21.7; 19.2; 18.6; MS (ESI): *m/z* = 421.2 ([M+Na]^+^); HRMS(ESI): *m/z* calcd. for C_19_H_34_N_4_O_3_SNa^+^ ([M+Na]^+^): 421.2244, found: 421.2239.

**N**_**2**_**=Phe-****d****-LeuΨ[4Tz]Val-OAll (6b):** (*R*)-Bus-d-Leu Ψ[4Tz]Val-OAll (3b, 74 mg, 0.19 mol) was dissolved in MeOH (2 mL). After addition of 4 M HCl in dioxane (0.14 mL) the mixture was stirred 2 h at rt. After evaporation of the solvent the product was used for the next step without further purification. ^**1**^**H-NMR** (500 MHz, CDCl_3_): δ [ppm] = 8.45 (s, 3H); 8.13 (s, 1H); 5.89 (m, 1H); 5.33 (dddd, ^4^*J* = 1.3 Hz, 1.3 Hz, ^3^*J* = 17.2 Hz, ^2^*J* = 1.2 Hz, 1H); 5.28 (dddd, ^4^*J* = 1.2 Hz, 1.2 Hz, ^3^*J* = 10.3 Hz, ^2^*J* = 1.0 Hz, 1H); 5.14 (d, ^3^*J* = 8.4 Hz, 1H); 4.72–4.61 (m, 3H); 2.50 (m, 1H); 2.00 (m, 1H); 1.92 (m, 1H); 1.50 (m, 1H); 0.99 (d, ^3^*J* = 6.7 Hz, 3H); 0.90 (d, ^3^*J* = 6.5 Hz, 3H); 0.89 (d, ^3^*J* = 6.5 Hz, 3H); 0.81 (d, ^3^*J* = 6.7 Hz, 3H). H-d-Leu*[4Tz]*Val-OAll·HCl (81 mg, 0.19 mmol) was stirred with the N_2_ = Phe-OH (1b, 39 mg 0.21 mmol), HATU (106 mg, 0.279 mmol), HOAt (38 mg, 0.28 mmol), and DIPEA (282 μL, 1.66 mmol) according to **GP-2**. Purification via column chromatography (DCM/MeOH 50:1, R_f_ = 0.35) yielded the product as colorless solid. **Yield:** 34 mg (73 μmol, 39%); ^**1**^**H-NMR** (500 MHz, CDCl_3_): δ [ppm] = 7.68 (s, 1H); 7.24–7.18 (m, 5H); 6.67 (d, ^3^*J* = 8.7 Hz, 1H); 5.90 (m, 1H); 5.34 (dddd, ^4^*J* = 1.4 Hz, 1.4 Hz, ^3^*J* = 17.2 Hz, ^2^*J* = 1.1 Hz, 1H); 5.29 (dddd, ^4^*J* = 1.1 Hz, 1.1 Hz, ^3^*J* = 10.4 Hz, ^2^*J* = 1.0 Hz, 1H); 5.21 (m, 1H); 5.12 (d, ^3^*J* = 8.8 Hz, 1H); 4.72–4.62 (m, 2H); 4.15 (dd, ^3^*J* = 8.1 Hz, 4.2 Hz, 1H); 3.29 (dd, ^3^*J* = 4.7 Hz, ^2^*J* = 14.0 Hz, 1H); 2.96 (dd, ^3^*J* = 8.1 Hz, ^2^*J* = 14.0 Hz, 1H); 2.46 (m, 1H); 1.83–1.73 (m, 2H); 1.45 (m, 1H); 1.03 (d, ^3^*J* = 6.7 Hz, 3H), 0.93 (d, ^3^*J* = 6.6 Hz, 3H); 0.92 (d, ^3^*J* = 6.4 Hz, 3H); 0.82 (d, ^3^*J* = 6.7 Hz, 3H); ^13^C-NMR (126 MHz, CDCl_3_): δ [ppm] = 168.4; 168.0; 148.1; 136.2; 131.0; 129.5; 128.8; 127.3; 120.8; 120.0; 69.0; 66.7; 65.5; 44.1; 44.0; 38.7; 32.6; 25.0; 22.7; 22.4; 19.3; 18.7; MS(ESI): *m/z* = 490.2 ([M+Na]^+^); HRMS(ESI): *m/z* calcd. for (C_24_H_33_N_7_O_3_)_2_Na^+^ ([2M+Na]^+^): 957.5182, found: 957.5158.

**(*R*)-Bus-****d****-LeuΨ[4Tz]Phe-****d****-LeuΨ[4Tz]Val-OAll (7b):** N_2_ = Phe-d-LeuΨ[4Tz]Val-OAll (6b, 34 mg, 73 μmol), the alkyne **(*R*)-Bus-****d****-Leu-CH** (2c, 17 mg, 79 μmol), CuSO_4_·5H_2_O (9.0 mg, 36 μmol) and sodium ascorbate (14 mg, 71 μmol) were stirred according to **GP-1**. Purification via preparative RP-HPLC yielded the product as colorless solid. Yield: 39 mg (56 μmol, 77%); Analytical RP-HPLC: *t*_*R*_ = 3.60 min (Method A1); Preparative RP-HPLC: *t*_*R*_ = 42.6 min (Method P2, 220 nm); ^1^H-NMR (500 MHz, CDCl_3_): δ [ppm] = 7.72 (s, 1H); 7.69 (s, 1H); 7.22–7.20 (m, 3H); 7.09–7.08 (m, 2H); 6.84 (d, ^3^*J* = 8.1 Hz, 1H); 5.90 (m, 1H); 5.34 (dd, ^3^*J* = 8.4 Hz, 6.9 Hz, 1H) 5.33 (dddd, ^4^*J* = 1.4 Hz, 1.4 Hz, ^3^*J* = 17.1 Hz, ^2^*J* = 1.3 Hz, 1H); 5.28 (dddd, ^4^*J* = 1.2 Hz, 1.2 Hz, ^3^*J* = 10.5 Hz, ^2^*J* = 1.2 Hz, 1H); 5.19 (m, 1H); 5.10 (d, ^3^*J* = 8.8 Hz, 1H); 4.71–4.61 (m, 2H); 4.79 (m, 1H); 3.62 (s *(br)*, 1H); 3.58 (dd, ^3^*J* = 6.9 Hz, ^2^*J* = 14.0 Hz, 1H); 2.96 (dd, ^3^*J* = 8.7 Hz, ^2^*J* = 13.9 Hz, 1H); 2.47 (m, 1H); 1.84 (m, 1H); 1.75 (m, 1H); 1.69–1.58 (m, 2H); 1.32–1.24 (m, 2H); 1.19 (s, 9H); 1.01 (d, ^3^*J* = 6.7 Hz, 3H), 0.91 (d, ^3^*J* = 7.0 Hz, 3H); 0.90 (d, ^3^*J* = 6.7 Hz, 3H); 0.85 (d, ^3^*J* = 6.5 Hz, 3H); 0.84 (d, ^3^*J* = 6.6 Hz, 3H); 0.80 (d, ^3^*J* = 6.7 Hz, 3H); ^13^C-NMR (126 MHz, CDCl_3_): δ [ppm] = 168.5; 166.8; 150.4; 148.3; 135.6; 131.1; 129.2; 128.9; 127.4; 122.3; 120.9; 119.9; 68.9; 66.7, 66.0; 56.4; 51.8; 45.2; 44.8; 43.6; 39.2; 32.6; 24.8; 24.6; 22.9; 22.8; 22.7; 22.3; 21.9; 19.3; 18.7; MS (ESI): *m/z* = 705.4 ([M+Na]^+^); HRMS (ESI): *m/z* calcd. for C_35_H_54_N_8_O_4_SNa^+^ ([M+Na]^+^): 705.38809, found: 705.38774.

**N**_**2**_**=Phe-****d****-LeuΨ[4Tz]Phe-****d****-LeuΨ[4Tz]Val-OAll (8b):** (*R*)-Bus-d-LeuΨ[4Tz]Phe-d-LeuΨ[4Tz]Val-OAll (15 mg, 22 μmol) was dissolved in MeOH (2 mL). After addition of 4 M HCl in dioxane (11 μL solution) the mixture was stirred 2 h at rt. After evaporation of the solvent the free amine was dissolved in DMF and stirred with the azido N_2_ = Phe-OH (1b, 5.0 mg, 26 μmol), HATU (13 mg, 34 μmol), HOAt (4.0 mg, 29 μmol), and DIPEA (34 μl, 0.20 mmol) according to **GP-2**. Purification via preparative RP-HPLC yielded the product as colorless solid. Yield: 9.0 mg (12 μmol, 54%); Preparative RP-HPLC: *t*_*R*_ = 28.2 min (Method P1, 220 nm); ^1^H-NMR (500 MHz, DMSO-*d*_6_): δ [ppm] = 8.96 (d, ^3^*J* = 8.6 Hz, 1H); 8.53 (d, ^3^*J* = 8.7 Hz, 1H); 8.16 (s, 1H); 8.01 (s, 1H); 7.32–7.18 (m, 10H); 5.87 (m, 1H); 5.61 (dd, ^3^*J* = 8.6 Hz, 6.7 Hz, 1H); 5.27 (d, ^3^*J* = 8.4 Hz, 1H); 5.23 (dddd, ^4^*J* = 1.4 Hz, 1.4 Hz, ^3^*J* = 17.3 Hz, ^2^*J* = 1.5 Hz, 1H); 5.19 (dddd, ^4^*J* = 1.3 Hz, 1.3 Hz, ^3^*J* = 10.6 Hz, ^2^*J* = 1.5 Hz, 1H); 5.03–4.96 (m, 2H); 4.67–4.60 (m, 2H); 3.95 (dd, ^3^*J* = 7.9 Hz, 6.7 Hz, 1H); 3.39 (dd, ^3^*J* = 6.7 Hz, ^2^*J* = 13.5 Hz, 1H); 3.34 (dd, ^3^*J* = 8.2 Hz, ^2^*J* = 13.5 Hz, 1H); 3.07 (dd, ^3^*J* = 6.8 Hz, ^2^*J* = 13.5 Hz, 1H); 2.98 (dd, ^3^*J* = 8.2 Hz, ^2^*J* = 13.5 Hz, 1H); 2.54 (m, 1H); 1.57 (m, 2H); 1.48–1.45 (m, 2H); 1.25 (m, 1H); 1.10 (m, 1H); 0.92 (d, ^3^*J* = 6.7 Hz, 3H), 0.82–0.78 (m, 9H); 0.75 (d, ^3^*J* = 6.5 Hz, 3H); 0.72 (d, ^3^*J* = 6.7 Hz, 3H); ^13^C-NMR (126 MHz, DMSO-*d*_6_): δ [ppm] = 168.1; 167.9; 165.5; 148.1; 147.8; 136.6; 135.8; 133.7; 129.1; 129.1; 128.3; 128.2; 126.; 126.7; 122.0; 121.3; 118.5; 67.5; 65.7; 63.7; 62.1; 43.7; 43.6; 43.5; 43.3; 39.5; 37.0; 30.6; 24.1; 23.9; 22.7; 22.5; 21.9; 21.8; 18.8; 18.2; MS(ESI): *m/z* = 774.4 ([M+Na]^+^).

**(*R*)-Bus-****d****-ValΨ[4Tz]Phe-****d****-LeuΨ[4Tz]Phe-****d****-LeuΨ[4Tz]Val-OAll (9b):** N_2_ = Phe-d-LeuΨ[4Tz]Phe-d-LeuΨ[4Tz]Val-OAll (8b, 24 mg, 32 μmol), the alkyne (*R*)-Bus-d-Val-CH (9.0 mg, 45 μmol) CuSO_4_·5H_2_O (4.0 mg, 16 μmol) and sodium ascorbate (6.0 mg, 30 μmol) were stirred according to **GP-1**. Purification via preparative RP-HPLC yielded the product as colorless solid. Yield: 17 mg (18 μmol, 56%); Analytical RP-HPLC: *t*_*R*_ = 3.74 min (Method A1); Preparative RP-HPLC: *t*_*R*_ = 46.2 min (Method P2, 220 nm); ^1^H-NMR (600 MHz, DMSO-*d*_6_): δ [ppm] = 8.90 (d, ^3^*J* = 8.6 Hz, 1H); 8.85 (d, ^3^*J* = 8.6 Hz, 1H); 8.21 (s, 1H); 8.11 (s, 1H); 7.98 (s, 1H); 7.23–7.15 (m, 10H); 5.87 (m, 1H); 5.62–5.56 (m, 2H); 5.26 (d, ^3^*J* = 8.4 Hz, 1H); 5.23 (dddd, ^4^*J* = 1.5 Hz, 1.5 Hz, ^3^*J* = 17.2 Hz, ^2^*J* = 1.6 Hz, 1H); 5.20 (s *(br)*, 1H); 5.18 (dddd, ^4^*J* = 1.5 Hz, 1.5 Hz, ^3^*J* = 10.6 Hz, ^2^*J* = 1.2 Hz, 1H); 5.01–4.96 (m, 2H); 4.67–4.60 (m, 2H); 4.11 (dd, ^3^*J* = 6.5 Hz, 1H); 3.39–3.27 (m, 4H); 2.52 (m, 1H); 2.05 (m, 1H); 1.57–1.54 (m, 2H); 1.54–1.47 (m, 2H); 1.24 (m, 1H); 1.17 (m, 1H); 1.12 (s, 9H); 0.91 (d, ^3^*J* = 6.7 Hz, 3H); 0.82–0.78 (m, 15H); 0.75 (d, ^3^*J* = 6.7 Hz, 3H); 0.72 (d, ^3^*J* = 6.7 Hz, 3H); ^13^C-NMR (151 MHz, DMSO-*d*_6_): δ [ppm] = 167.9; 166.5; 166.5; 148.3; 148.1; 147.6; 135.8; 135.8; 131.7; 129.1; 129.0; 128.2; 128.2; 126.7; 126.7; 122.2; 122.0; 121.3; 118.; 67.5; 65.7; 63.7; 63.7; 58.3; 55.7; 43.8; 43.6; 43.5; 43.4; 38.4; 37.9; 33.3; 30.6; 24.1; 24.0; 22.7; 22.6; 22.5; 21.9; 21.8; 19.0; 18.8; 18.3; 18.2; MS(ESI): *m/z* = 975.5 ([M+Na]^+^); HRMS(ESI): *m/z* calcd. for C_35_H_54_N_8_O_4_SNa^+^ ([M+Na]^+^): 975.5362, found: 975.5332.

**Boc-Ala-****d****-ValΨ[4Tz]Phe-****d****-LeuΨ[4Tz]Phe-****d****-LeuΨ[4Tz]Val-OAll (10b):** (*R*)-Bus-d-ValΨ[4Tz]Phe-d-LeuΨ[4Tz]Phe-d-LeuΨ[4Tz]Val-OAll (9b, 23.3 mg, 25.0 μmol) is dissolved in 1 mL of a 1:1 mixture of DCM and HCl (4 M in dioxane) and stirred for 2 h at rt. After complete cleavage of the Boc-group, the free amine is evaporated to dryness. In a second flask, Boc-Ala-OH (9.5 mg, 50 μmol) and HOAt (7.5 mg, 55 μmol) are dissolved in DCM (2 mL, dry). After addition of DIC (8.5 μL, 55 μmol), the mixture is stirred 5 min at RT for preactivation and is then added to the free amine, followed by TMP (16.5 μL; 125 μmol). This solution is left stirring over night at rt. After evaporation of the solvent, the crude product is purified by preparative HPLC. Yield: 15.4 mg (15.1 μmol, 60%); Analytical RP-HPLC: *t*_*R*_ = 6.60 min (Method A2); ^1^H-NMR (600 MHz, DMSO-*d*_6_): δ [ppm] = 8.92 (d, *J* = 8.6 Hz, 1H), 8.89 (d, *J* = 8.7 Hz) 8.19 (s, 1H), 8.14 (s, 1H), 8.00 (s, 1H), 7.88 (d, *J* = 9.2 Hz), 7.23–7.14 (m, 10H) 6.89 (d, *J* = 7.6 Hz, 1H), 5.86 (ddt, *J* = 17.2, 10.6, 5.5 Hz, 1H), 5.63–5.58 (m, 2H), 5.26 (d, *J* = 8.4 Hz, 1H), 5.23 (dd, *J* = 17.2, 1.7 Hz, 1H), 5.18 (dd, *J* = 10.6, 1.5 Hz, 1H), 5.01–4.96 (m, 2H), 4.81 (dd, *J* = 9.2, 6.4 Hz, 1H), 4.64 (m, 2H), 4.03 (dq, *J* = 7.6, 7.2 Hz, 1H), 3.40–3.26 (m, 4H), 2.50 (m, 1H), 2.05–1.96 (m, 1H), 1.58–1.50 (m, 4H), 1.36 (s, 9H), 1.28–1.14 (m, 2H), 1.18 (d, *J* = 7.2 Hz), 0.91 (d, *J* = 6.7 Hz, 3H), 0.82–0.78 (m, 12H), 0.75 (d, *J* = 6.7 Hz, 3H), 0.72 (d, *J* = 6.8 Hz, 3H), 0.70 (d, *J* = 6.8 Hz, 3H); ^13^C-NMR (151 MHz, DMSO-*d*_6_): δ [ppm] = 172.77, 168.32, 166.96, 166.94, 155.42, 148.53, 148.04, 147.33, 136.27, 136.22, 132.16, 129.50, 129.48, 128.62, 127.20, 122.46, 122.28, 121.82, 118.91, 78.49, 67.96, 66.14, 64.17, 50.82, 50.38, 44.21, 44.05, 43.90, 38.81, 38.41, 32.80, 31.08, 28.63, 24.56, 24.46, 23.09, 22.97, 22.37, 22.26, 19.34,19.24, 18.84, 18.68, 18.41; HRMS (ESI): m/z calcd. for C_54_H_78_N_13_${\text{O}}_{7}^{+}$ ([M+H]^+^): 1020.6142, found: 1020.6126.

**Boc-GlyΨ[4Tz]Val-OAll (3c):** N_2_ = Val-OAll (104 mg, 0.568 mmol), the alkyne Boc-Gly-CH (2e, 100 mg, 0.644 mmol), CuSO_4_·5H_2_O (71 mg, 0.29 mmol) and sodium ascorbate (112 mg, 0.565 mmol) were stirred according to **GP-1**. Purification via column chromatography (PE/EE 2:1, *R*_*f*_ = 0.54) yielded the product as colorless solid. Yield: 138 mg (0.408 mmol, 72%);${\left[\alpha \right]}_{D}^{20}$ = 40.8 (c = 0.7, CHCl_3_); ^1^H-NMR (500 MHz, CDCl_3_): δ [ppm] = 7.86 (s, 1H); 5.90 (m, 1H,); 5.34 (dddd, ^4^*J* = 1.5 Hz, 1.5 Hz, ^3^*J* = 17.2 Hz, ^2^*J* = 1.2 Hz, 1H); 5.29 (dddd, ^4^*J* = 1.5 Hz, 1.5 Hz, ^3^*J* = 10.4 Hz, ^2^*J* = 1.0 Hz, 1H); 5.23 (s *(br)*, 1H); 5.12 (d, ^3^*J* = 8.6 Hz, 1H); 4.71–4.64 (m, 2H); 4.42 (d, ^3^*J* = 5.3 Hz, 2H); 2.49 (m, 1H); 1.43 (s, 9H); 1.02 (d, ^3^*J* = 6.7 Hz, 3H); 0.83 (d, ^3^*J* = 6.7 Hz, 3H); ^13^C-NMR (126 MHz, CDCl_3_): δ [ppm] = 168.3; 156.0; 145.5; 131.0; 122.0; 120.0; 80.0; 69.2; 66.8; 36.0; 32.5; 28.5; 19.3; 18.6; MS (ESI): *m/z* = 361.2 ([M+Na]^+^).

**N**_**2**_**=Phe-GlyΨ[4Tz]Val-OAll (6c):** Boc-GlyΨ[4Tz]Val-OAll (3c, 63 mg, 0.19 mmol) was dissolved in TFA (2 mL) and stirred for 12 h. After evaporation of TFA in vacuo the crude product was used for the next step without further purification. ^1^H-NMR (500 MHz, CDCl_3_): δ [ppm] = 8.62 (s, 3H); 8.16 (s, 1H); 5.89 (m, 1H); 5.33 (dddd, ^4^*J* = 1.4 Hz, 1.4 Hz, ^3^*J* = 17.2 Hz, ^2^*J* = 1.3 Hz, 1H); 5.27 (dddd, ^4^*J* = 1.3 Hz, 1.3 Hz, ^3^*J* = 10.4 Hz, ^2^*J* = 1.0 Hz, 1H); 5.14 (d, ^3^*J* = 8.5 Hz, 1H); 4.71–4.60 (m, 2H); 4.37 (s (br), 2H); 2.50 (m, 1H); 0.99 (d, ^3^*J* = 6.7 Hz, 3H); 0.81 (d, ^3^*J* = 6.7 Hz, 3H); ^13^C-NMR (126 MHz, CDCl_3_): δ [ppm] = 168.2; 161.9 (q); 140.3; 131.0; 124.2; 120.0; 116.3 (q); 69.2; 66.9; 34.8; 32.3; 19.1; 18.3. H-GlyΨ[4Tz]Val-OAll·TFA (55 mg, 0.16 mmol) and the azido acid N_2_ = Phe-OH (1b, 34 mg, 0.18 mmol) were stirred with HATU (93 mg, 0.24 mmol), HOAt (33 mg, 0.24 mmol) and DIPEA (0.25 mL, 1.5 mmol) according to **GP-2**. Purification via preparative RP-HPLC yielded the product as colorless solid. Yield: 34 mg (83 μmol, 52% over two steps); Preparative RP-HPLC: *t*_*R*_ = 23.8 min (Method P1, 220 nm); ^1^H-NMR (500 MHz, CDCl_3_): δ [ppm] = 7.82 (s, 1H); 7.29–7.21 (m, 5H); 7.01 (dd, ^3^*J* = 5.2 Hz, 5.2 Hz, 1H); 5.91 (m, 1H); 5.35 (dddd, ^4^*J* = 1.3 Hz, 1.3 Hz, ^3^*J* = 17.2 Hz, ^2^*J* = 1.2 Hz, 1H); 5.30 (dddd, ^4^*J* = 1.2 Hz, 1.2 Hz, ^3^*J* = 10.4 Hz, ^2^*J* = 1.1 Hz, 1H); 5.13 (d, ^3^*J* = 8.6 Hz, 1H); 4.72–4.64 (m, 2H); 4.56–4.48 (m, 2H); 4.19 (dd, ^3^*J* = 8.3 Hz, 4.4 Hz, 1H); 3.32 (dd, ^3^*J* = 4.4 Hz, ^2^*J* = 14.0 Hz, 1H); 3.00 (dd, ^3^*J* = 8.3 Hz, ^2^*J* = 14.0 Hz, 1H); 2.49 (m, 1H); 1.03 (d, ^3^*J* = 6.7 Hz, 3H); 0.85 (d, ^3^*J* = 6.7 Hz, 3H); ^13^C-NMR (126 MHz, CDCl_3_): δ [ppm] = 169.0; 168.2; 144.0; 136.1; 130.9; 129.6; 128.8; 127.4; 122.3; 120.1; 69.2; 66.8; 65.5; 38.7; 34.8; 32.5; 19.3; 18.7; MS (ESI): *m/z* = 434.2 ([M+Na]^+^).

**Boc-GlyΨ[4Tz]Phe-GlyΨ[4Tz]Val-OAll (7c):** N_2_ = Phe-GlyΨ[4Tz]Val-OAll (6c, 34 mg, 83 μmol) was stirred with Boc-Gly-CH (2e, 19 mg, 0.12 mmol), CuSO_4_·5H_2_O (10 mg, 0.040 mmol) and sodium ascorbate (16 mg, 81 μmmol) according to **GP-1**. Purification via preparative RP-HPLC yielded the product as colorless solid. Yield: 19 mg (34 μmol, 41%); Analytical RP-HPLC: *t*_*R*_ = 3.04 min (Method A1); Preparative RP-HPLC: *t*_*R*_ = 32.9 min (Method P2, 220 nm); ^1^H-NMR (600 MHz, CDCl_3_): δ [ppm] = 7.74 (s, 1H); 7.59 (s, 1H); 7.22–7.19 (m, 3H); 7.03–7.02 (m, 2H); 6.99 (dd, ^3^*J* = 5.8 Hz, 5.8 Hz, 1H); 5.89 (m, 1H); 5.34 (dddd, ^4^*J* = 1.5 Hz, 1.5 Hz, ^3^*J* = 17.2 Hz, ^2^*J* = 1.4 Hz, 1H); 5.29 (dddd, ^4^*J* = 1.3 Hz, 1.3 Hz, ^3^*J* = 10.4 Hz, ^2^*J* = 1.3 Hz, 1H); 5.27 (dd, ^3^*J* = 8.6 Hz, 6.9 Hz, 1H); 5.11 (d, ^3^*J* = 8.6 Hz, 1H); 5.11 (s *(br)*, 1H); 4.70 (dddd, ^4^*J* = 1.3 Hz, 1.3 Hz, ^3^*J* = 6.0 Hz, ^2^*J* = 13.0 Hz, 1H); 4.65 (dddd, ^4^*J* = 1.3 Hz, 1.3 Hz, ^3^*J* = 6.0 Hz, ^2^*J* = 13.0 Hz, 1H); 4.74–4.64 (m, 2H); 4.35 (m, 2H); 3.56 (dd, ^3^*J* = 6.5 Hz, ^2^*J* = 13.9 Hz, 1H); 3.38 (dd, ^3^*J* = 8.9 Hz, ^2^*J* = 13.9 Hz, 1H); 2.47 (m, 1H); 1.43 (s, 9H); 1.03 (d, ^3^*J* = 6.7 Hz, 3H); 0.83 (d, ^3^*J* = 6.7 Hz, 3H); ^13^C-NMR (151 MHz, CDCl_3_): δ [ppm] = 168.4; 167.4; 155.9; 145.7; 143.9; 135.4; 131.0; 129.0; 128.9; 127.6; 122.4; 122.0; 120.1; 79.9; 69.1; 66.8; 66.3; 39.5; 36.2; 35.5; 32.6; 28.5; 19.3; 18.8; MS (ESI): *m/z* = 589.3 ([M+Na]^+^); HRMS (ESI): *m/z* calcd. for C_19_H_34_N_4_O_3_SNa^+^ ([M+Na]^+^): 589.2857, found: 589.2852.

**N**_**2**_**=Phe-GlyΨ[4Tz]Phe-GlyΨ[4Tz]Val-OAll (8c):** Boc-GlyΨ[4Tz]Phe-GlyΨ[4Tz]Val-OAll (7c, 78 mg, 0.138 mmol) was dissolved in TFA (2 mL) and stirred for 18 h. After evaporation of TFA in vacuo the free amine, the azido carboxylic acid N_2_ = Phe-OH (1b, 39 mg, 0.21 mmol), HATU (79 mg, 0.20 mmol), HOAt (28 mg, 0.21 mmol) and DIPEA (0.21 mL, 1.2 mmol) were stirred according to **GP-2**. Purification via preparative RP-HPLC yielded the product as colorless solid. Yield: 38 mg (59 μmol, 43%); Preparative RP-HPLC: *t*_*R*_ = 34.2 min (Method P3, 254 nm); ^1^H-NMR (500 MHz, CDCl_3_): δ [ppm] = 7.75 (s, 1H); 7.58 (s, 1H); 7.48 (dd, ^3^*J* = 5.6 Hz, 5.6 Hz, 1H); 7.28–7.16 (m, 8H); 7.08 (dd, ^3^*J* = 5.1 Hz, 5.1 Hz, 1H); 7.03–7.01 (m, 2H); 5.88 (m, 1H); 5.38 (dd, ^3^*J* = 8.4 Hz, 7.1 Hz, 1H); 5.32 (dddd, ^4^*J* = 1.5 Hz, 1.5 Hz, ^3^*J* = 17.1 Hz, ^2^*J* = 1.5 Hz, 1H); 5.28 (dddd, ^4^*J* = 1.3 Hz, 1.3 Hz, ^3^*J* = 10.4 Hz, ^2^*J* = 1.3 Hz, 1H); 5.09 (d, ^3^*J* = 8.6 Hz, 1H); 4.70–4.60 (m, 2H); 4.74–4.46 (m, 2H); 4.43–4.38 (m, 2H); 4.16 (dd, ^3^*J* = 8.4 Hz, 4.4 Hz, 1H); 3.55 (dd, ^3^*J* = 6.8 Hz, ^2^*J* = 13.9 Hz, 1H); 3.35 (dd, ^3^*J* = 8.7 Hz, ^2^*J* = 13.9 Hz, 1H); 3.29 (dd, ^3^*J* = 4.4 Hz, ^2^*J* = 14.0 Hz, 1H); 2.97 (dd, ^3^*J* = 8.4 Hz, ^2^*J* = 14.0 Hz, 1H); 2.47 (m, 1H); 1.01 (d, ^3^*J* = 6.7 Hz, 3H); 0.81 (d, ^3^*J* = 6.7 Hz, 3H); ^13^C-NMR (126 MHz, CDCl_3_): δ [ppm] = 169.0; 168.3; 167.4; 144.2; 143.9; 136.3; 135.3; 131.0; 129.6; 129.0; 128.9; 128.8; 127.5; 127.3; 122.8; 122.2; 120.0; 69.1; 66.8; 65.9; 65.3; 39.4; 38.6; 35.3; 34.9; 32.5; 19.3; 18.7; MS(MALDI): *m/z* = 640.3 ([M+H]^+^); 662.3 ([M+Na]^+^); 678.3 ([M+K]^+^).

**Boc-GlyΨ[4Tz]Phe-GlyΨ[4Tz]Phe-GlyΨ[4Tz]Val-OAll (9c):** N_2_ = Phe-GlyΨ[4Tz]Phe-GlyΨ[4Tz]Val-OAll (8c, 38 mg, 59 μmol) was stirred with the alkyne Boc-Gly-CH (2e, 14 mg, 88 μmol), CuSO_4_·5H_2_O (7.4 mg, 30 μmol) and sodium ascorbate (11.7 mg, 59.0 μmol) according to **GP-1**. Purification via preparative RP-HPLC yielded the product as colorless solid. Yield: 7.0 mg (9 μmol, 15%); Analytical RP-HPLC: *t*_*R*_ = 3.12 min (Method A1); Preparative RP-HPLC: *t*_*R*_ = 34.0 min (Method P3, 220 nm); ^1^H-NMR (500 MHz, DMSO-*d*_6_): δ [ppm] = 9.05 (dd, ^3^*J* = 5.5 Hz, 5.5 Hz, 1H); 8.99 (dd, ^3^*J* = 5.6 Hz, 5.6 Hz, 1H); 8.07 (s, 1H); 8.05 (s, 1H); 7.94 (s, 1H); 7.28 (dd, ^3^*J* = 6.0 Hz, 6.0 Hz, 1H); 7.22–7.14 (m, 10H); 5.90 (m, 1H); 5.67–5.57 (m, 2H); 5.31 (d, ^3^*J* = 8.2 Hz, 1H); 5.28 (dddd, ^4^*J* = 1.5 Hz, 1.5 Hz, ^3^*J* = 17.2 Hz, ^2^*J* = 1.5 Hz, 1H); 5.23 (dddd, ^4^*J* = 1.4 Hz, 1.4 Hz, ^3^*J* = 10.5 Hz, ^2^*J* = 1.4 Hz, 1H); 4.70–4.62 (m, 2H); 4.41–4.34 (m, 2H); 4.33–4.26 (m, 2H); 4.14 (m, 2H); 3.41–3.30 (m, 4H); 2.55 (m, 1H); 1.30 (s, 9H); 0.95 (d, ^3^*J* = 6.7 Hz, 3H); 0.77 (d, ^3^*J* = 6.7 Hz, 3H); ^13^C-NMR (126 MHz, DMSO-*d*_6_): δ [ppm] = 167.9; 167.4; 167.3; 155.6; 145.3; 143.8; 143.6; 136.2; 136.0; 131.8; 128.9; 128.9; 128.3; 128.3; 126.8; 126.8; 123.1; 122.2; 121.9; 118.6; 77.9; 67.5; 65.8; 63.7; 63.7; 37.8; 37.6; 35.6; 34.4; 34.4; 30.7; 28.2; 18.8; 18.3; MS(ESI): *m/z* = 817.5 ([M+Na]^+^); HRMS(ESI): *m/z* calcd. for C_40_H_50_N_12_O_6_Na^+^ ([M+Na]^+^): 817.3868, found: 817.3874.

**Boc-Ala-GlyΨ[4Tz]Phe-GlyΨ[4Tz]Phe-GlyΨ[4Tz]Val-OAll (10c):** Boc-GlyΨ[4Tz]Phe-GlyΨ[4Tz]Phe-GlyΨ[4Tz]Val-OAll (9c, 50 mg, 63 μmol) is dissolved in 2 mL of a 1:1 mixture of DCM and HCl (4 M in dioxane) and stirred for 2 h at rt. After complete cleavage of the Boc-group, the free amine is evaporated to dryness. In a second flask, Boc-Ala-OH (24 mg, 0.13 mmol) and HOAt (19 mg, 0.14 mmol) are dissolved in DCM (5 mL, dry). After addition of DIC (22 μL, 0.14 mmol), the mixture is stirred 5 min at RT for preactivation and is then added to the free amine, followed by TMP (42 μL, 0.32 mmol). This solution is left stirring over night at rt. After evaporation of the solvent, the crude product is purified by preparative HPLC. Yield: 41 mg (47 μmol, 75%); Analytic RP-HPLC: *t*_*R*_ = 5.3 min (Method A2); ^1^H-NMR (600 MHz, DMSO-*d*_6_): δ [ppm] = 9.04 (dd, *J* = 5.61, 5.57 Hz, 1H), 9.00 (dd, *J* = 5.56, 5.43 Hz, 1H), 8.26 (dd, *J* = 5.99, 5.93 Hz, 1H), 8.08 (s, 1H), 8.04 (s, 1H), 7.94 (s, 1H), 7.25–7.08 (m, 10H), 6.91 (d, J = 7.4 Hz, 1H), 5.94–5.85 (m, 1H), 5.62–5.57 (m, 2H), 5.30 (d, *J* = 8.4 Hz, 1H), 5.28 (dd, *J* = 17.2, 1.7 Hz, 1H), 5.23 (dd, *J* = 10.5, 1.6 Hz, 1H), 4.70–4.62 (m, 2H), 4.41–4.24 (m, 6H), 3.97 (dq, 1H, *J* = 7.4, 7.2 Hz), 3.42–3.27 (m, 4H) 2.54 (dqq, *J* = 8.4, 6.7, 6.5 Hz, 1H), 1.38 (s, 9H), 1.17 (d, *J* = 7.2 Hz, 3H), 0.94 (d, *J* = 6.5 Hz, 3H), 0.77 (d, *J* = 6.7 Hz, 3H); ^13^C-NMR (151 MHz, DMSO-*d*_6_): δ [ppm] = 173.15, 168.33, 167.73, 155.51, 145.26, 144.25, 144.03, 136.52, 136.49, 132.21, 129.25, 128.24, 127.24, 123.49, 122.66, 122.40, 119.06, 78.44, 67.98, 66.23, 64.16, 50.13, 38.29, 38.08, 34.84, 34.83, 34.80, 31.12, 28.68, 19.26, 18.71; HRMS (ESI): m/z calcd. for C_43_H_56_N_13_${\text{O}}_{7}^{+}$ ([M+H]^+^): 866.4420, found: 866.4413.

### Conformational Analysis

Molecular dynamics (MD) simulations were performed on a compute cluster located at the Center for Biotechnology (CeBiTec), Bielefeld University, using the CUDA accelerated version of the pmemd program as implemented in AMBER (Amber 16, AmberTools 17) (Case et al., 2017). All simulations were based on the additional force-field TZLff (Marion et al., 2018) parametrized for 1,4- and 1,5- disubstituted peptidotriazolamers.

Identical simulation strategies were applied for the conformational analysis of all molecules. First, 100 parallel simulated annealing (SA) runs were performed in the gas phase by applying NMR-derived inter-proton restraints (Supplementary Tables 1–3). No cutoff was applied on non-bonded interactions and the temperature ramp was set to gradually heat up the molecule from 0 to 600 K and finally cool it down to 0 K over a 1 ns-long MD run(10 K, 5 ps; 100 K, 5 ps; 200 K, 90 ps; 600 K, 400 ps; 400 K, 100 ps; 200 K, 200 ps; 100 K, 75 ps; 10 K, 20 ps; 0 K, 5 ps). Interproton distances were calculated from ROESY-NMR spectra using equation 3 by Ämmälahti et al. (1996). The harmonic restraints were applied only to the upper bound, with a force constant of 32 kcal·mol^−1^·Å^−2^ (as set in the input of AMBER). After an initial series of 100 runs, NMR restraint restraints causing penalties <5 kcal·mol^−1^, were regarded as potential artifacts from the spectrum interpretation and removed from the set of restraints. The procedure was then repeated until no restraint passed the penalty ceiling.

The representative structure of the main cluster (based on backbone atoms only) among the SA replica was further selected as a starting structure for MD simulations in solvent. Each molecule was solvated in a truncated octaedron box of (i) TIP4Pew water (Horn et al., 2004) molecules and (ii) DMSO (Fox and Kollman, 1998) molecules, the solvent used for NMR, with a 15 Å buffer region. After minimization, the systems were gradually heated up, first in the NVT ensemble from 0 to 100 K, and then in the NPT ensemble from 100 to 300 K over a total of 890 ps (10 K, 10 ps; 50 K, 10 ps; 100 K, 20 ps; 100 K, 50 ps; 200 K, 100 ps; 300 K, 200 ps; 300 K, 500 ps). Production runs were then performed in the NPT ensemble for 200 ns. All simulations of solvated systems used a time step of 2 fs, periodic boundary conditions with particle mesh Ewald and a non-bonded cutoff of 12 Å, and the SHAKE algorithm to constrain the length of all bonds involving a hydrogen atom. Temperature and pressure were controlled with Langevin dynamics (collision frequency of 2.0 ps^−1^) and isotropic position scaling, respectively. For each system, one simulation in water and one simulation in DMSO were performed without applying any NMR restraint.

The simulations for the longer poly-alanine mimics (poly-Ala) followed the same protocol and were performed without restraints in water and in DMSO. For each poly-Ala system, four parallel simulations were ran starting from the same coordinates after heatup but with different initial velocities in order to increase the sampling of the conformational space and ensure convergence of our analysis.

Analysis of the trajectories were performed using cpptraj module of AmberTools17 (Roe and Cheatham, 2013). The output data was post processed using python 2 including NumPy and matplotlib modules (Hunter, 2007; Oliphant, 2007). Cluster analysis was performed using cpptraj's hierarchical agglomerative approach with a total of 10 clusters based on backbone RMSD (N, C^O^, C^α^, Ψ[4Tz]). Root mean square deviation (RMSD) and radius of gyration (RoG) analysis were performed based on backbone atoms only (N, C^O^, C^α^, Ψ[4Tz]) using the heatup structure as reference point in the case of RMSD. Dihedral analysis was done using the ϕ*-* and ψ- torsion angles defined in Figure 4. The torsion angle distribution was plotted in a Ramachandran like style using matplotlib. Radial distribution functions (RDFs) were calculated using a bin spacing of 0.1 Å and normalized using the default density of water as implemented in cpptraj. The representative structures of the main clusters were prepared using the VMD software (Humphrey et al., 1996).

## Data Availability

This manuscript contains previously unpublished data. The name of the repository and accession number are not available.

## Author Contributions

The project was designed, coordinated and supervised by NS. Synthesis of the peptidotriazolamers was performed by TF, OK and AN as well as the measurement of spectroscopic data by TF, OK, AN, and DS. Analysis of the NMR data for the use as distance restraints was done by DS, MJ, JG, under supervision of RL. The molecular modeling study was designed by AM and IA, supervised by AM, and executed mainly by DS, with assistance of MJ and JG. DS analyzed and compiled the data and prepared the manuscript with support of AM, NS, RL, and IA. The final manuscript was read and approved by all authors.

### Conflict of Interest Statement

The authors declare that the research was conducted in the absence of any commercial or financial relationships that could be construed as a potential conflict of interest.

## Supplementary Material

Supplementary Material as mentioned above as well as NMR data and PDB files of the representative structures are available online.
