# Supplementary material for: 1,4-Disubstituted 1H-1,2,3-Triazole Containing Peptidotriazolamers: A New Class of Peptidomimetics With Interesting Foldamer Properties
Source: Front Chem. 2019 Mar 26;7:155. doi: 10.3389/fchem.2019.00155 (PMC6443886; doi:10.3389/fchem.2019.00155)
Supplement: Supplementary file 1 [file Data_Sheet_1.PDF]

Supplementary Material

1,4-Disubstituted 1,2,3-Triazole-Containing Peptidotriazolamers: A New Class of Peptidomimetics with Interesting Foldamer Properties

David C. Schröder, Oliver Kracker, Tanja Fröhr, Jerzy Góra, Michał Jewginski, Anke Nieß, Iris Antes, Rafał Latajka, Antoine Marion* and Norbert Sewald*.

*** Correspondence:** Prof. Dr. Norbert Sewald: norbert.sewald@uni-bielefeld.de; Dr. Antoine Marion amarion@metu.edu.tr

TABLE OF CONTENTS

1 NMR Data 2

1.1 ^{1}H and ^{13}C spectra 2

1.2 ROESY derived inter proton distances 11

2 Computational Analysis 14

2.1 Representative heptameric peptidotriazolamers 10a-c 14

2.2 Homochiral, heterochiral, and homochiral* poly-alanine derivatives 18

1 NMR Data

1.1 ^{1}H and ^{13}C spectra

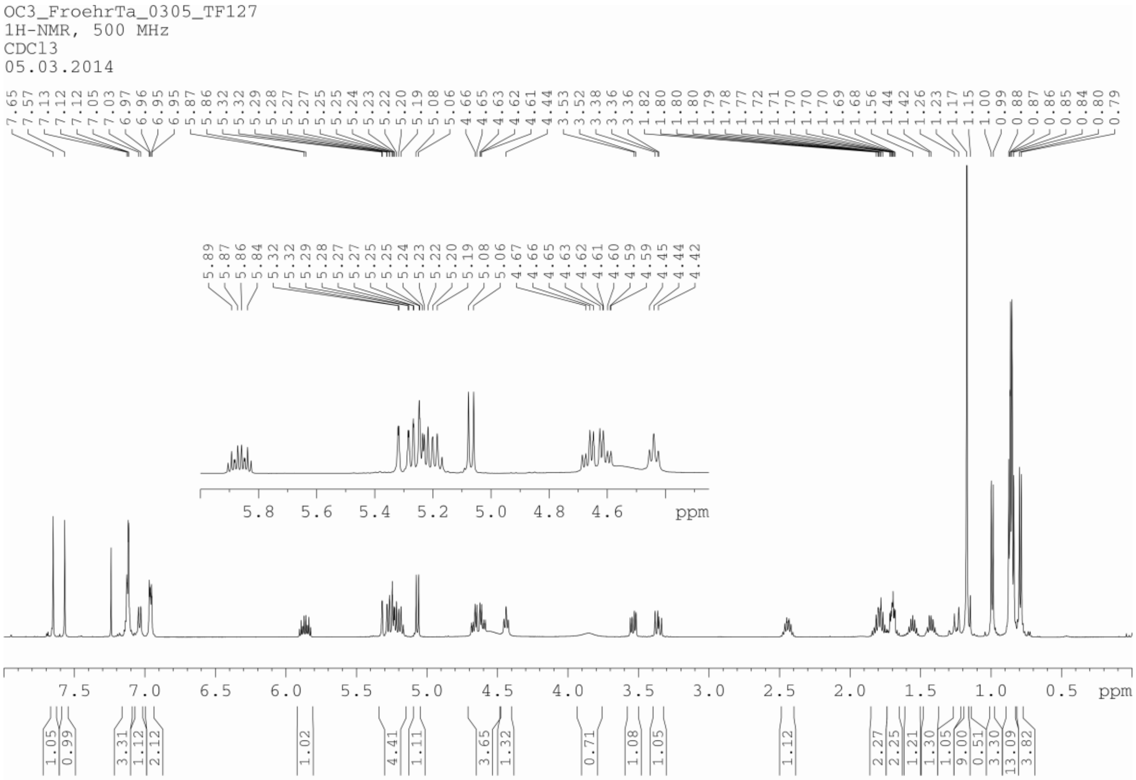

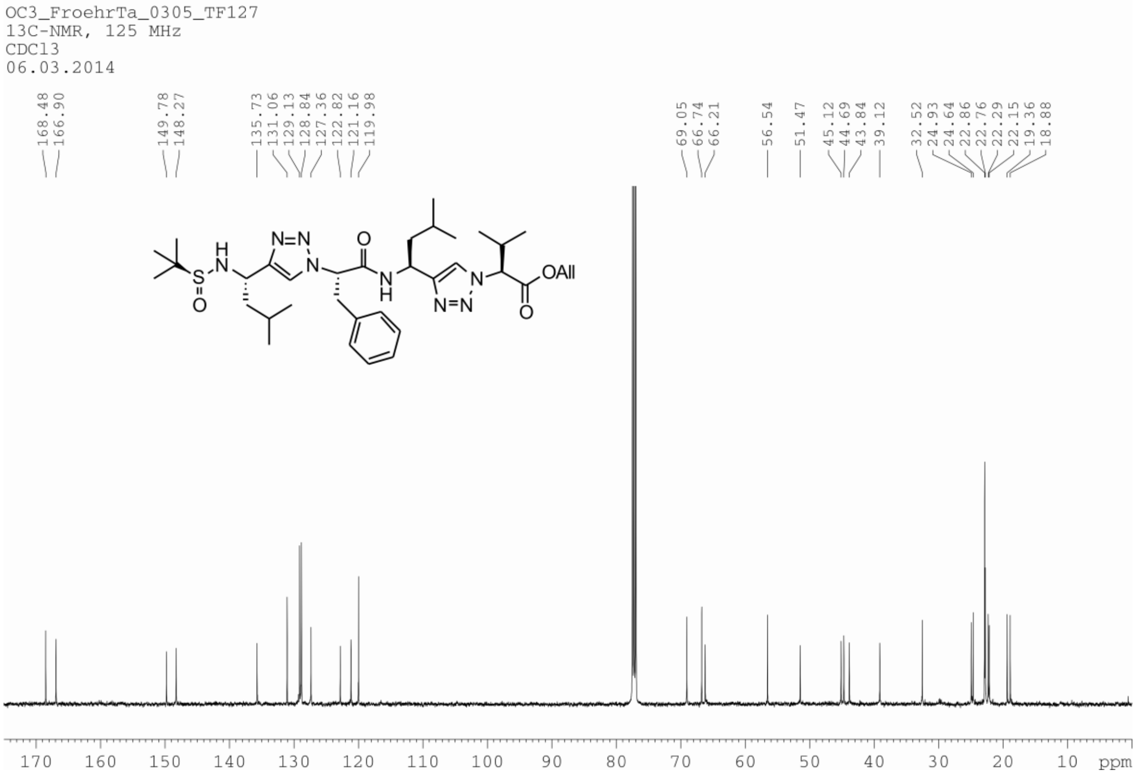

Supplementary Figure 1. ^{1}H- and ^{13}C-NMR spectra of 7a.

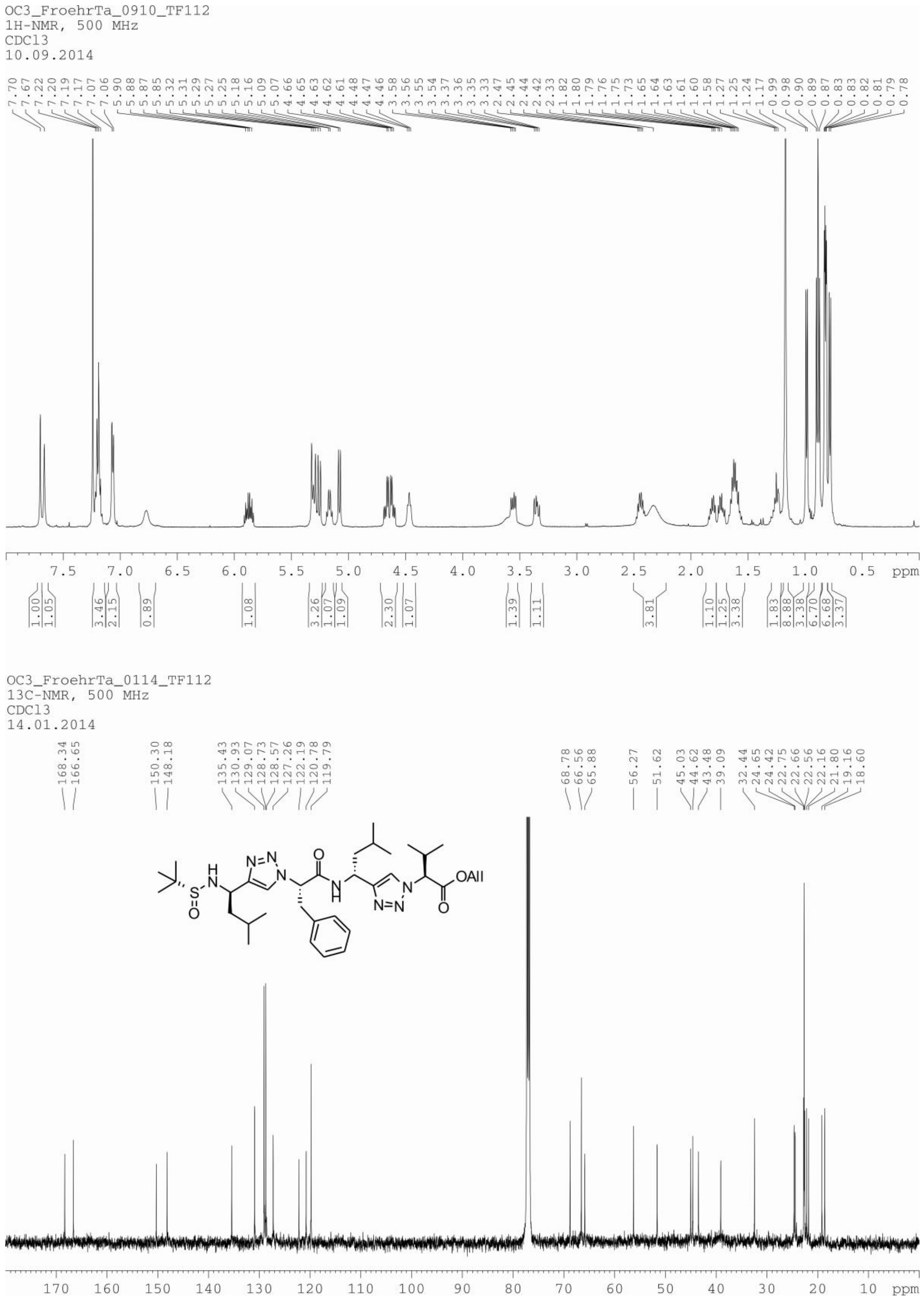

Supplementary Figure 2. ^{1}H- and ^{13}C-NMR spectra of 7b.

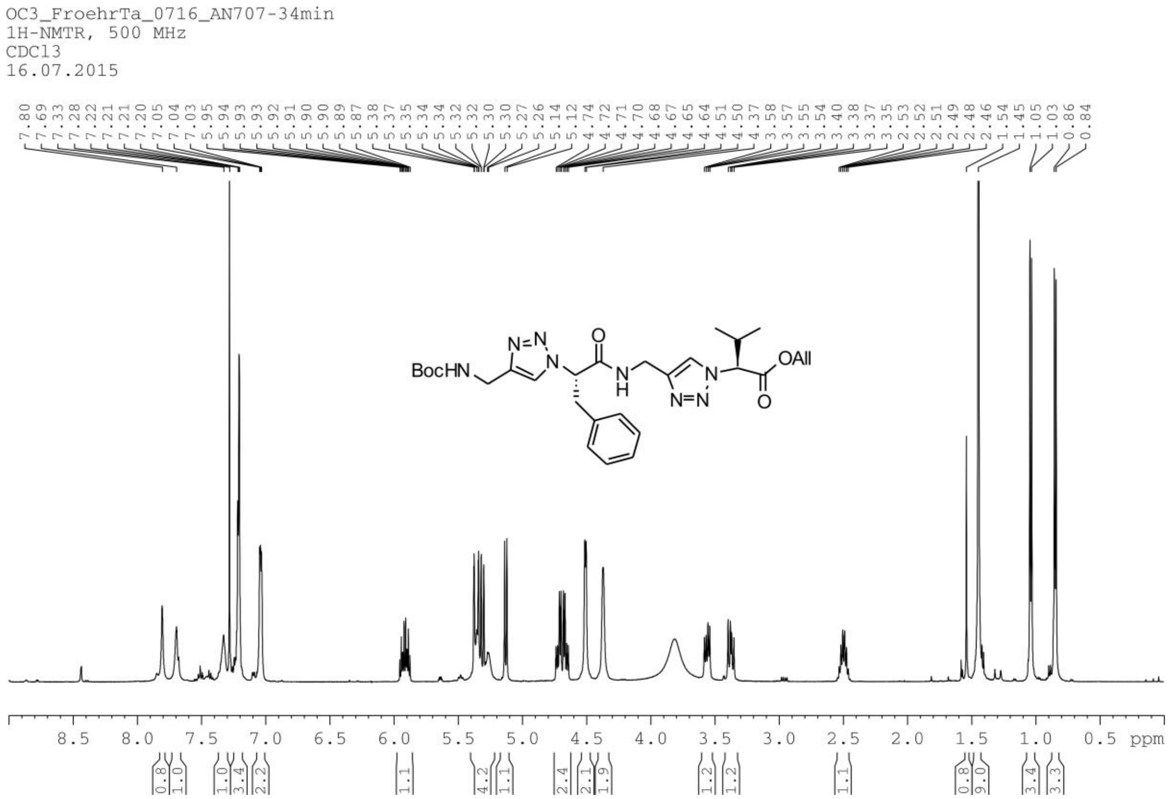

Supplementary Figure 3. ^{1}H- and ^{13}C-NMR spectra of 7c.

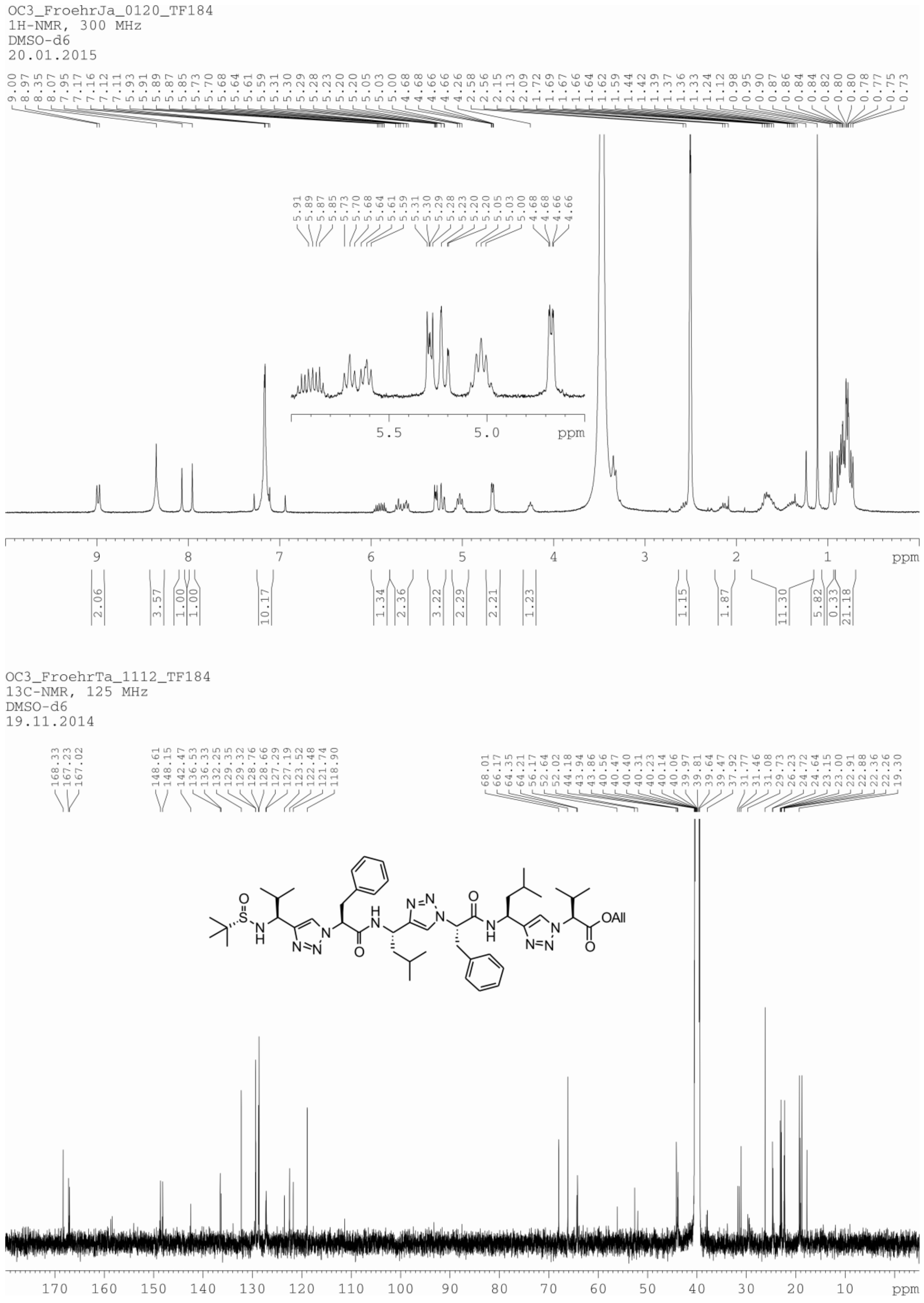

Supplementary Figure 4. ^{1}H- and ^{13}C-NMR spectra of 9a.

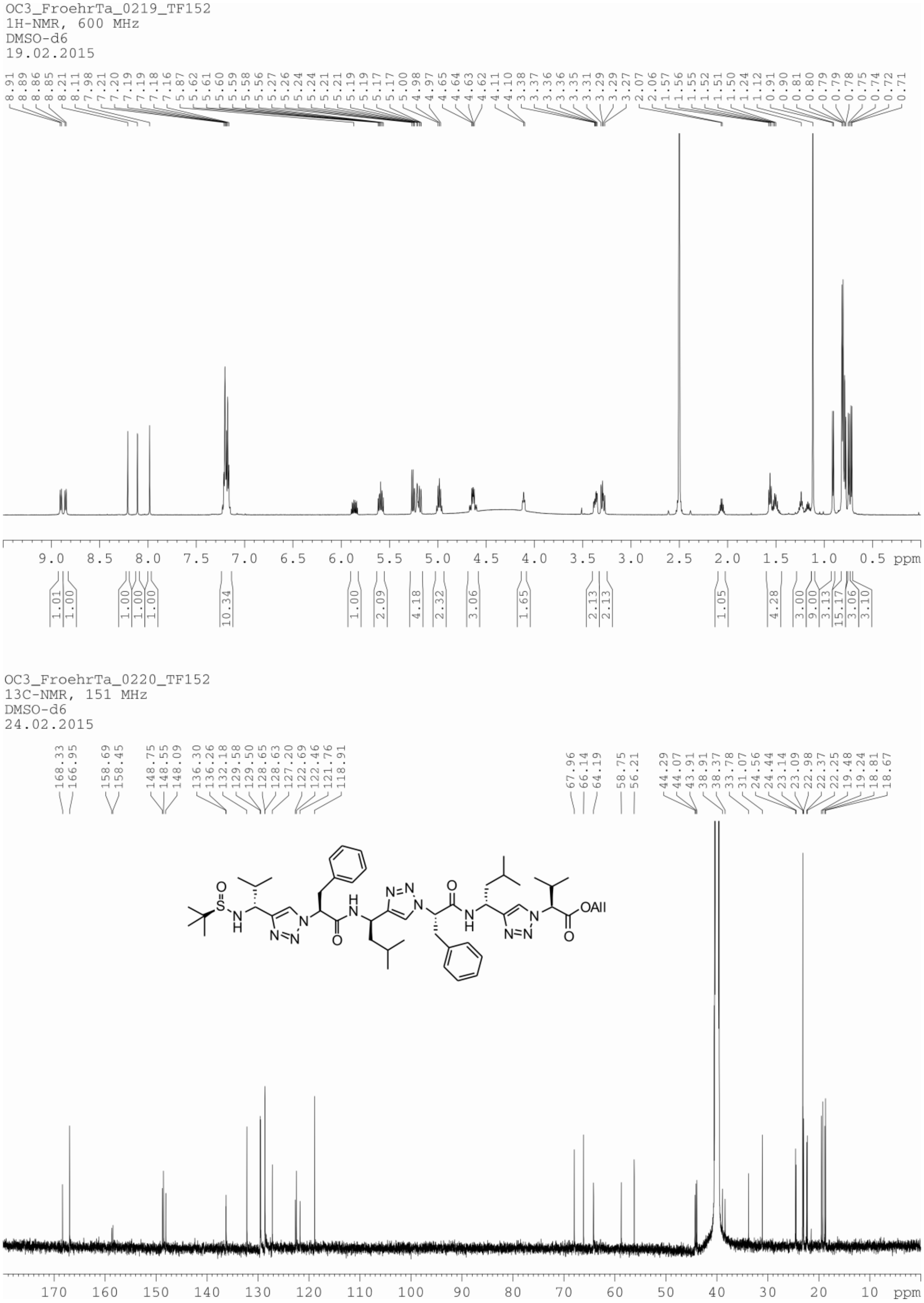

Supplementary Figure 5. ^{1}H- and ^{13}C-NMR spectra of 9b.

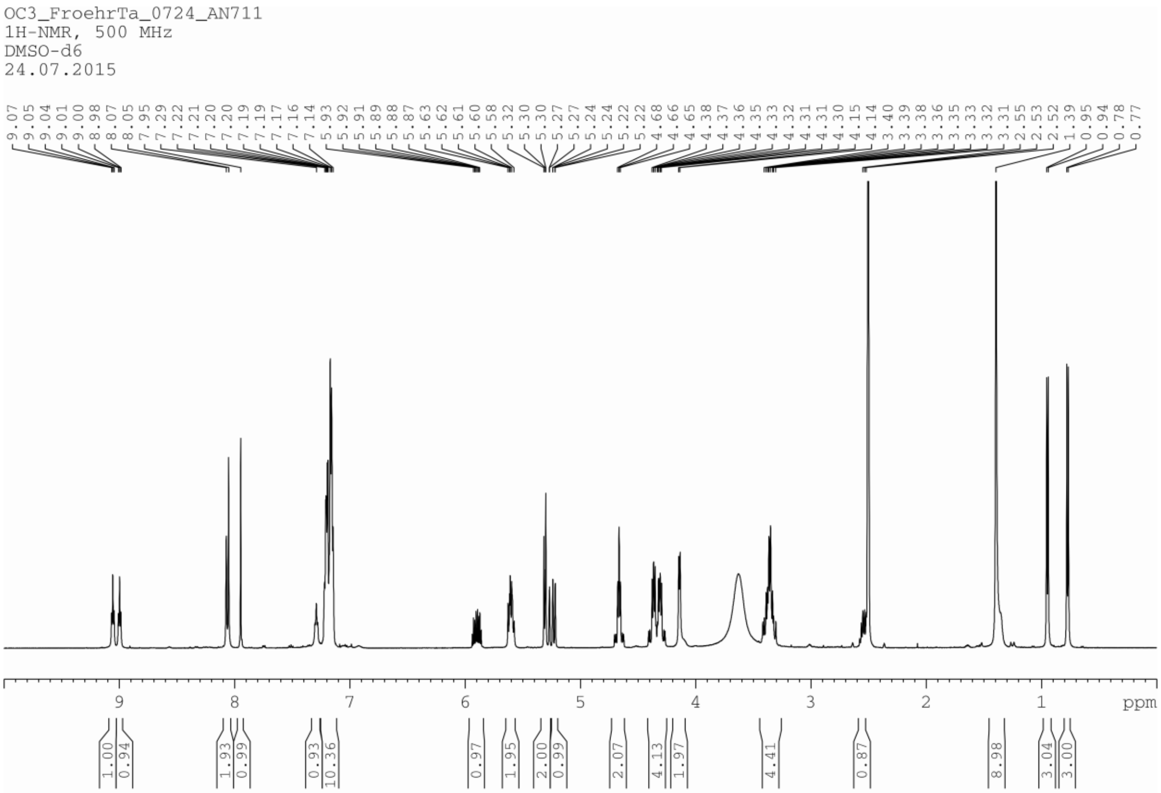

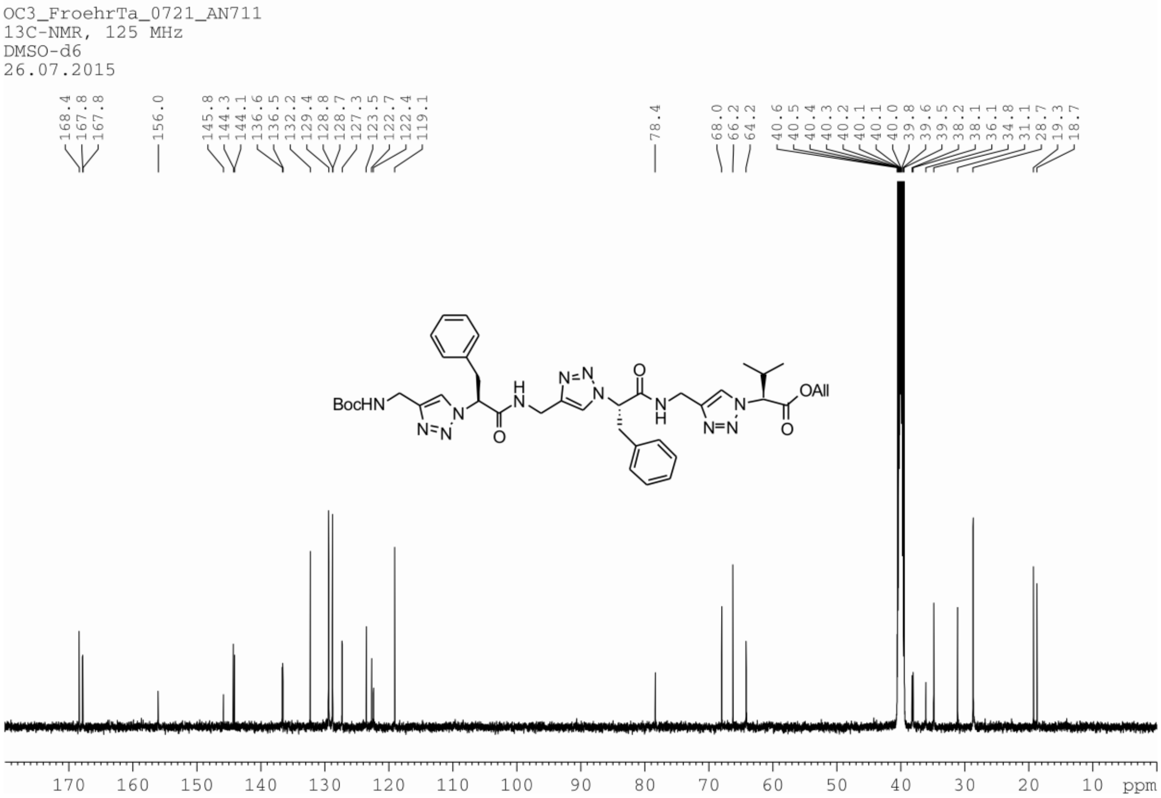

Supplementary Figure 6. ^{1}H- and ^{13}C-NMR spectra of 9c.

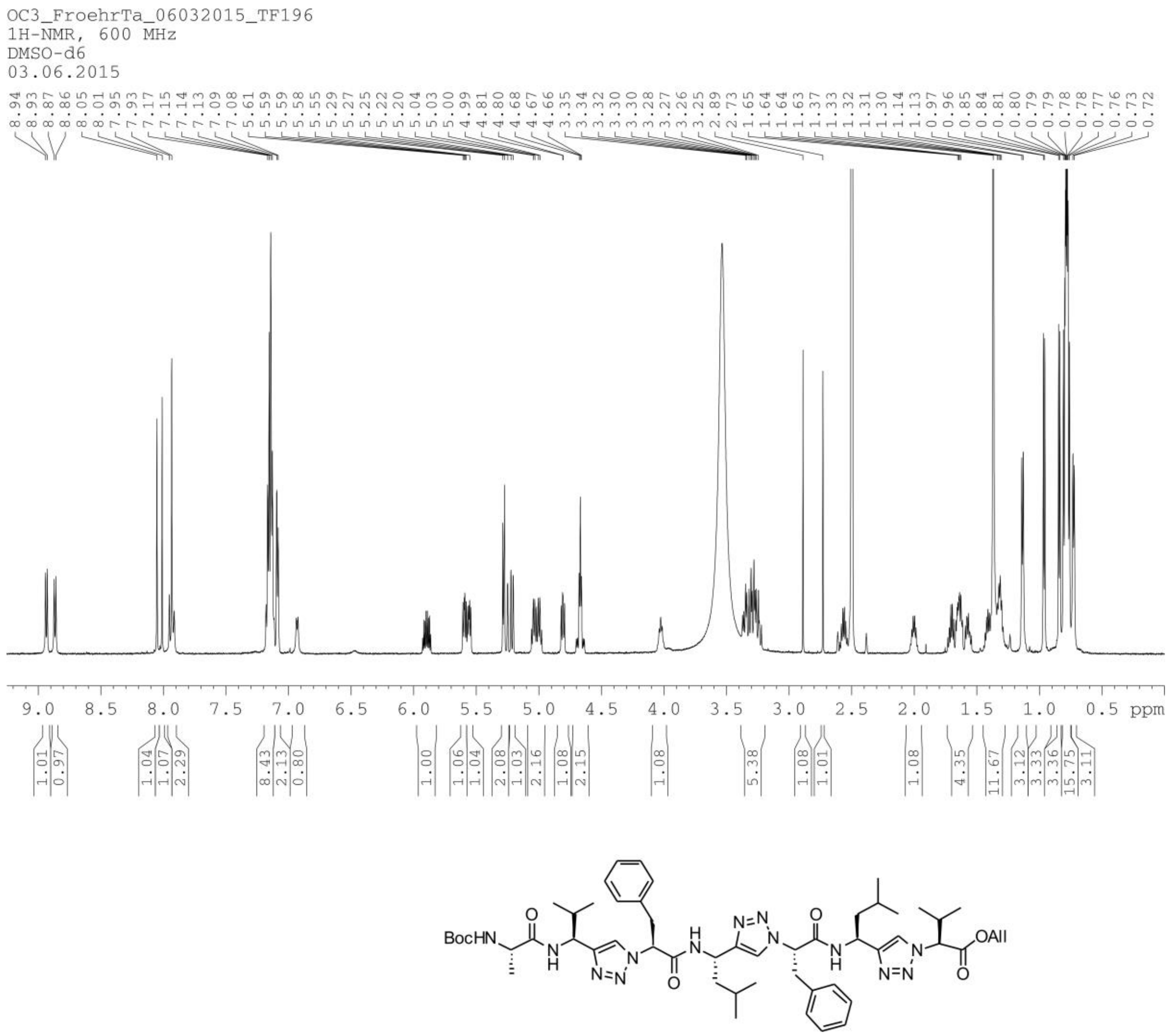

Supplementary Figure 7. ^{1}H- spectrum of 10a.

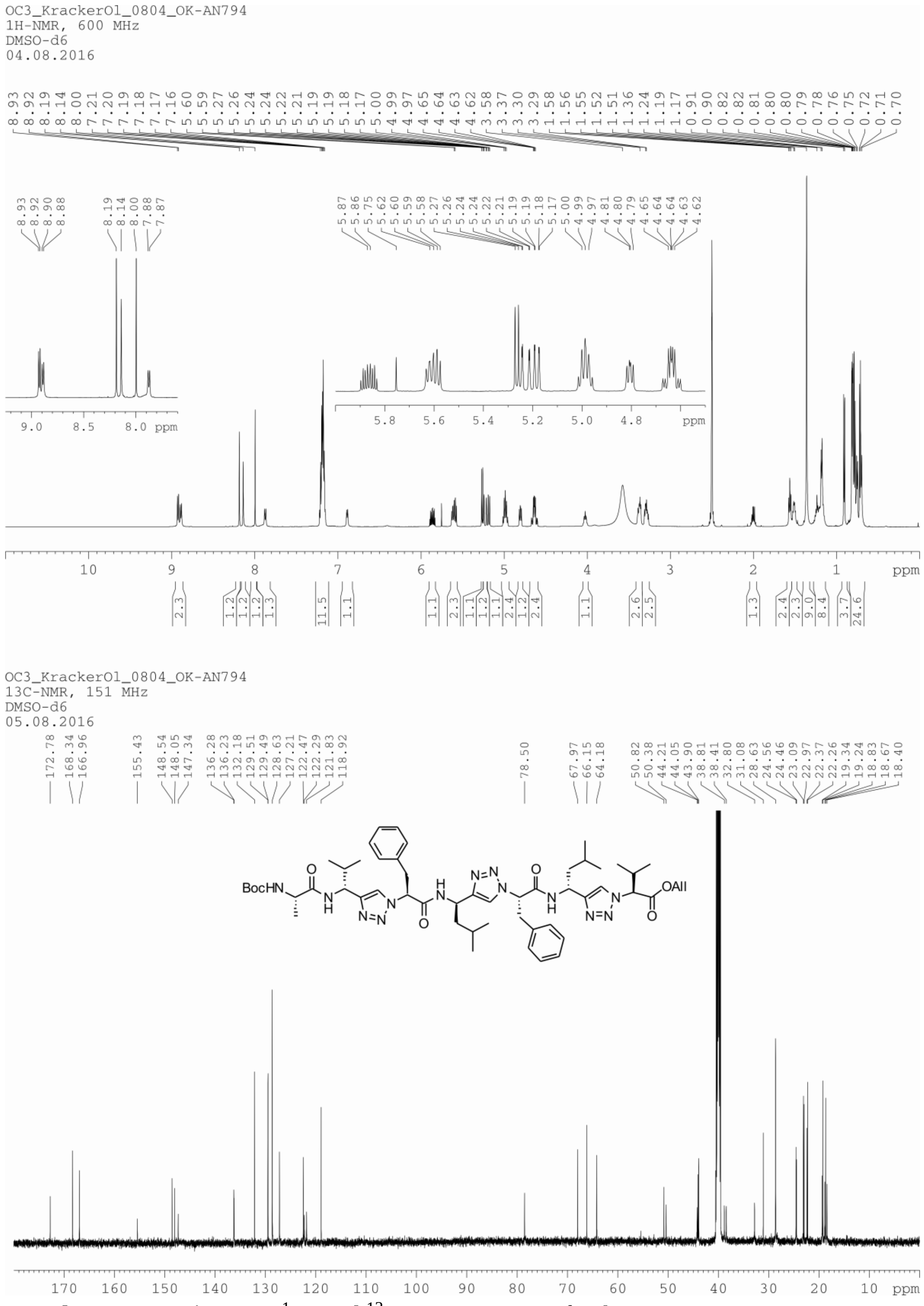

Supplementary Figure 8. ^{1}H- and ^{13}C-NMR spectra of 10b.

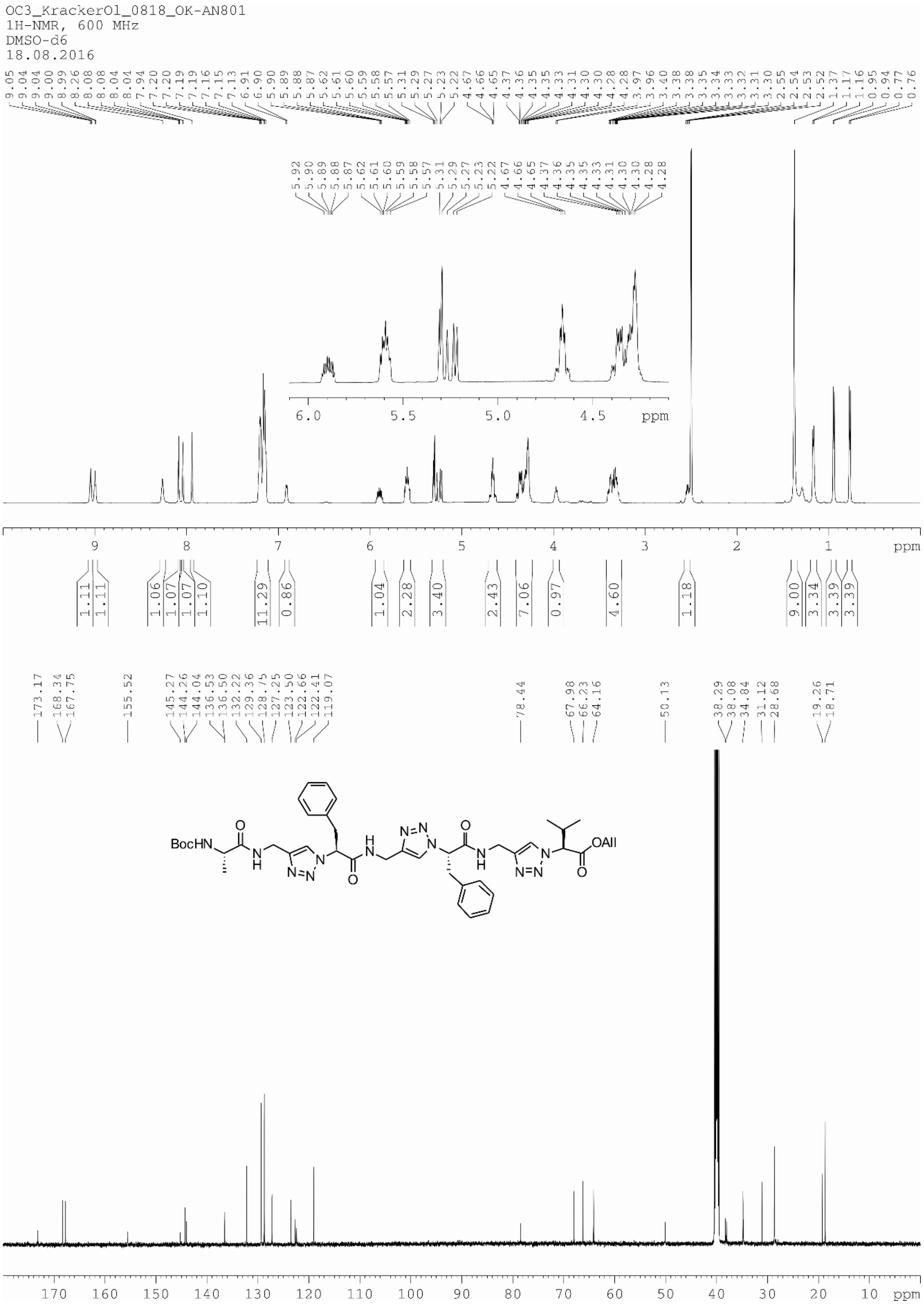

Supplementary Figure 9. ^{1}H- and ^{13}C-NMR spectra of 10c.

1.2 ROESY derived inter proton distances

Supplementary Table 1: ROESY-NMR derived inter-proton distances of 10a.

Residue 1	Atom 1	Residue 2	Atom 2	Distance /Å
Ala1	H	Ala1	HA	3.8
Ala1	HA	Ala1	H	3.7
Ala1	HA	Val2	H	2.7
Ala1	HB1 HB2 HB3*	Ala1	H	3.3
Ala1	HB1 HB2 HB3*	Ala1	HA	2.8
Ala1	HB1 HB2 HB3*	Val2	H	2.9
Ala1	HB1 HB2 HB3*	4Tz1	H5	4.3
Val2	H	Ala1	H	4.0
Val2	HA	Val2	H	2.9
Val2	HA	4Tz1	H5	3.3
Val2	HB	Val2	HA	2.7
Val2	HB	Val2	H	2.7
Val2	HB	4Tz1	H5	3.6
Val2	HG11 HG12 HG13*	Val2	HA	2.6
Val2	HG11 HG12 HG13*	Val2	HB	2.4
Val2	HG11 HG12 HG13*	4Tz1	H5	3.4
Phe3	HA	4Tz1	H5	3.4
Phe3	HA	Phe3	HD1 HD2*	2.9
Phe3	HA	Leu4	H	2.6
Phe3	HB2	4Tz1	H5	2.7
Phe3	HB2	Leu4	H	3.4
Phe3	HD1 HD2*	4Tz1	H5	3.8
Leu4	HA	Leu4	H	2.9
Leu4	HA	4Tz2	H5	3.1
Leu4	HB2 HB3*	Leu4	H	3.0
Leu4	HB2 HB3*	Leu4	HA	2.7
Leu4	HB2 HB3*	4Tz2	H5	3.1
Leu4	HD11-13 HD21-23*	Leu4	HA	2.8
Leu4	HD11-13 HD21-23*	4Tz2	H5	4.1
Leu4	HG	Leu4	H	3.7
Leu4	HG	Leu4	HA	3.5
Leu4	HG	4Tz2	H5	4.3
Phe5	HA	4Tz2	H5	3.3
Phe5	HA	Phe5	HD1 HD2*	2.9
Phe5	HA	Leu6	H	2.6
Phe5	HB2	4Tz2	H5	2.7

Phe5	HB2	Leu6	H	3.4
Phe5	HB2	4Tz3	H5	4.3
Phe5	HD1 HD2*	4Tz2	H5	3.6
Leu6	HA	Leu6	H	3.2
Leu6	HA	4Tz3	H5	3.2
Leu6	HB2 HB3*	Leu6	H	3.2
Leu6	HB2 HB3*	Leu6	HA	2.7
Leu6	HB2 HB3*	4Tz3	H5	3.6
Leu6	HD11-13 HD21-23*	Leu6	HA	2.8
Leu6	HD11-13 HD21-23*	4Tz3	H5	3.1
Leu6	HG	Leu6	H	3.8
Leu6	HG	Leu6	HA	3.5
Val7	HA	4Tz3	H5	3.1
Val7	HB	4Tz3	H5	3.1
Val7	HG11 HG12 HG13*	Val7	HA	2.4
Val7	HG11 HG12 HG13*	Val7	HB	2.8
Val7	HG21 HG22 HG23*	Val7	HA	2.6
Val7	HG21 HG22 HG23*	Val7	HB	2.8

Supplementary Table 2: ROESY-NMR derived inter-proton distances of 10b.

Residue 1	Atom 1	Residue 2	Atom 2	Distance /Å
Boc	H*	Ala1	H	4.0
Ala1	HA	Ala1	H	3.4
Ala1	HA	Val2	H	2.6
Ala1	HB1 HB2 HB3*	Ala1	HA	2.6
Ala1	HB1 HB2 HB3*	Ala1	H	3.1
Ala1	HB1 HB2 HB3*	Val2	HA	4.3
Ala1	HB1 HB2 HB3*	Val2	H	3.3
Val2	HA	4Tz1	H5	2.9
Val2	HA	Val2	H	3.0
Val2	HB	Val2	HG11 HG12 HG13*	2.4
Val2	HB	Val2	HG21 HG22 HG23*	2.4
Val2	HB	Val2	H	3.2
Val2	HB	4Tz1	H5	3.6
Val2	H	ALA	HB1 HB2 HB3*	3.4
Val2	H	Val2	HG11 HG12 HG13*	3.2
Val2	H	Val2	HG21 HG22 HG23*	3.7
4Tz1	H5	Val2	HG11 HG12 HG13*	3.6

4Tz1	H5	Val2	HG21 HG22 HG23*	3.9
Phe5	HA	4Tz2	H5	3.4
Phe3	HA	4Tz1	H5	3.3
4Tz3	H5	Val7	HG11 HG12 HG13*	3.5
4Tz3	H5	Val7	HG21 HG22 HG23*	4.2
Val7	HB	4Tz2	H5	5.1
Val7	HB	4Tz3	H5	2.9
Val7	HG11 HG12 HG13*	Val2	HA	2.6
Val7	HG11 HG12 HG13*	Val7	HA	3.1
Val7	HG21 HG22 HG23*	Val7	HA	2.9
Val7	HG21 HG22 HG23*	ALL	H11 H12*	4.2
ALL	H11 H12*	ALL	H21	3.1

Supplementary Table 3: ROESY-NMR derived inter-proton distances of 10c.

Residue 1	Atom 1	Residue 2	Atom 2	Distance /Å
Ala1	HA	Gly2	H	3.0
Ala1	HB1 HB2 HB3*	Ala1	H	3.4
Ala1	HB1 HB2 HB3*	Ala1	HA	2.8
Ala1	HB1 HB2 HB3*	Gly2	H	3.4
Gly2	HA2 HA3	Gly2	H	2.9
Gly2	HA2 HA3	4Tz1	H5	3.4
Phe3	HA	Gly4	H	2.6
Gly4	HA2 HA3*	Gly4	H	2.9
Gly4	HA2 HA3*	4Tz2	H5	2.9
Phe5	HA	Gly6	H	2.7
Gly6	HA2 HA3*	Gly6	H	2.9
Gly6	HA2 HA3*	4Tz3	H5	3.1
Val7	HA	4Tz3	H5	3.0
Val7	HB	4Tz3	H5	3.0
Val7	HB	Val7	HA	2.6
Val7	HG11 HG12 HG13*	4Tz3	H5	3.4
Val7	HG11 HG12 HG13*	Val7	HA	3.0
Val7	HG11 HG12 HG13*	Val7	HB	2.8
Val7	HG21 HG22 HG23*	Val7	HA	2.7
Val7	HG21 HG22 HG23*	Val7	HB	2.8
ALL	H11 H12*	ALL	H21	3.2

2 Computational Analysis

2.1 Representative heptameric peptidotriazolamers 10a-c

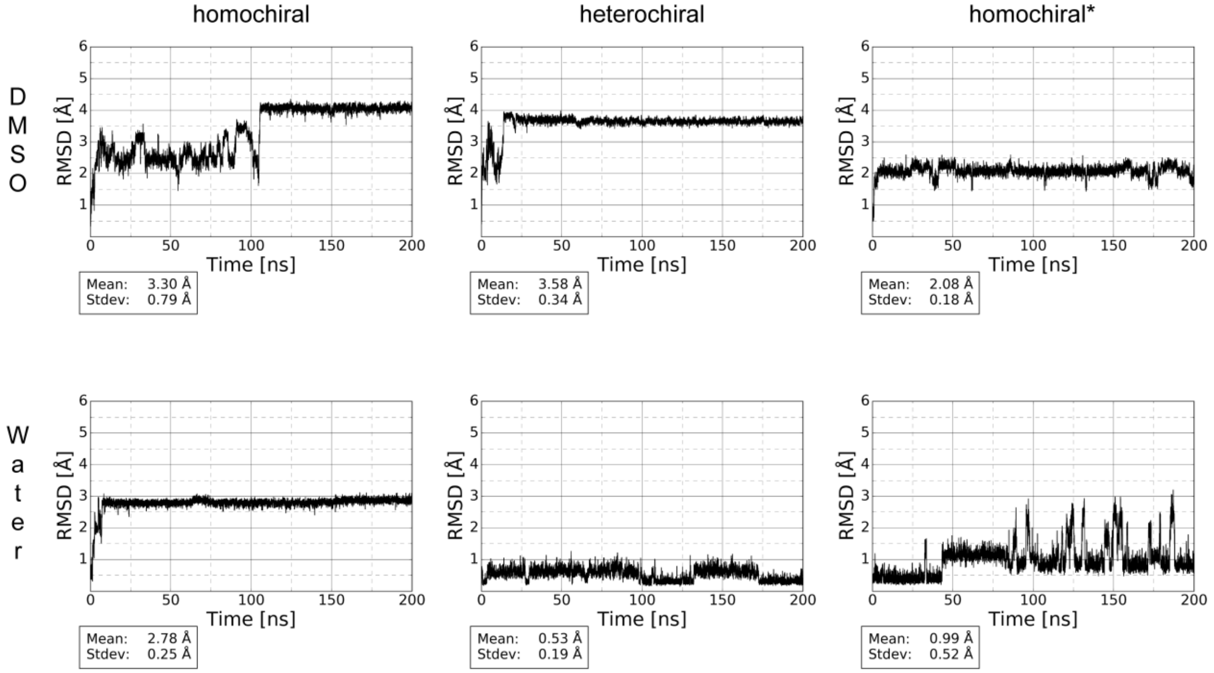

Supplementary Figure 10. Backbone RMSD time series for the whole trajectory of each molecule in DMSO (first row) and TIP4Pew water (second row). The starting point of the trajectory (after heat up) is taken as reference point.

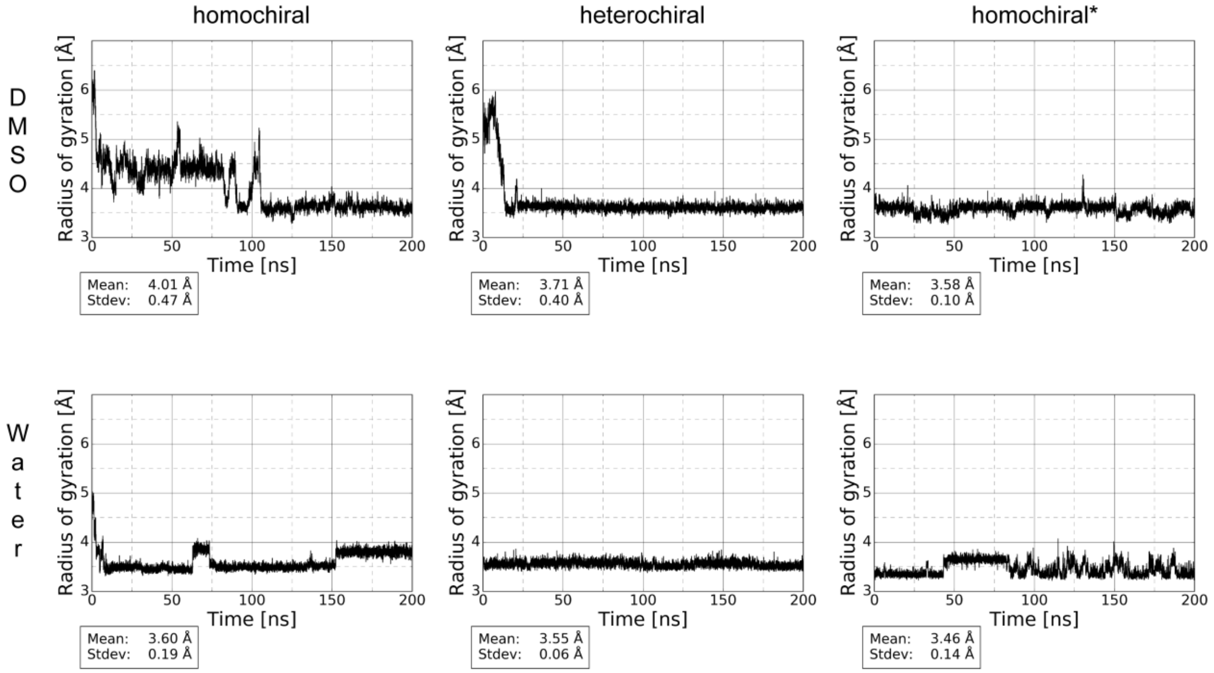

Supplementary Figure 11. Backbone radius of gyration time series for the whole trajectory of each molecule in DMSO (first row) and TIP4Pew water (second row).

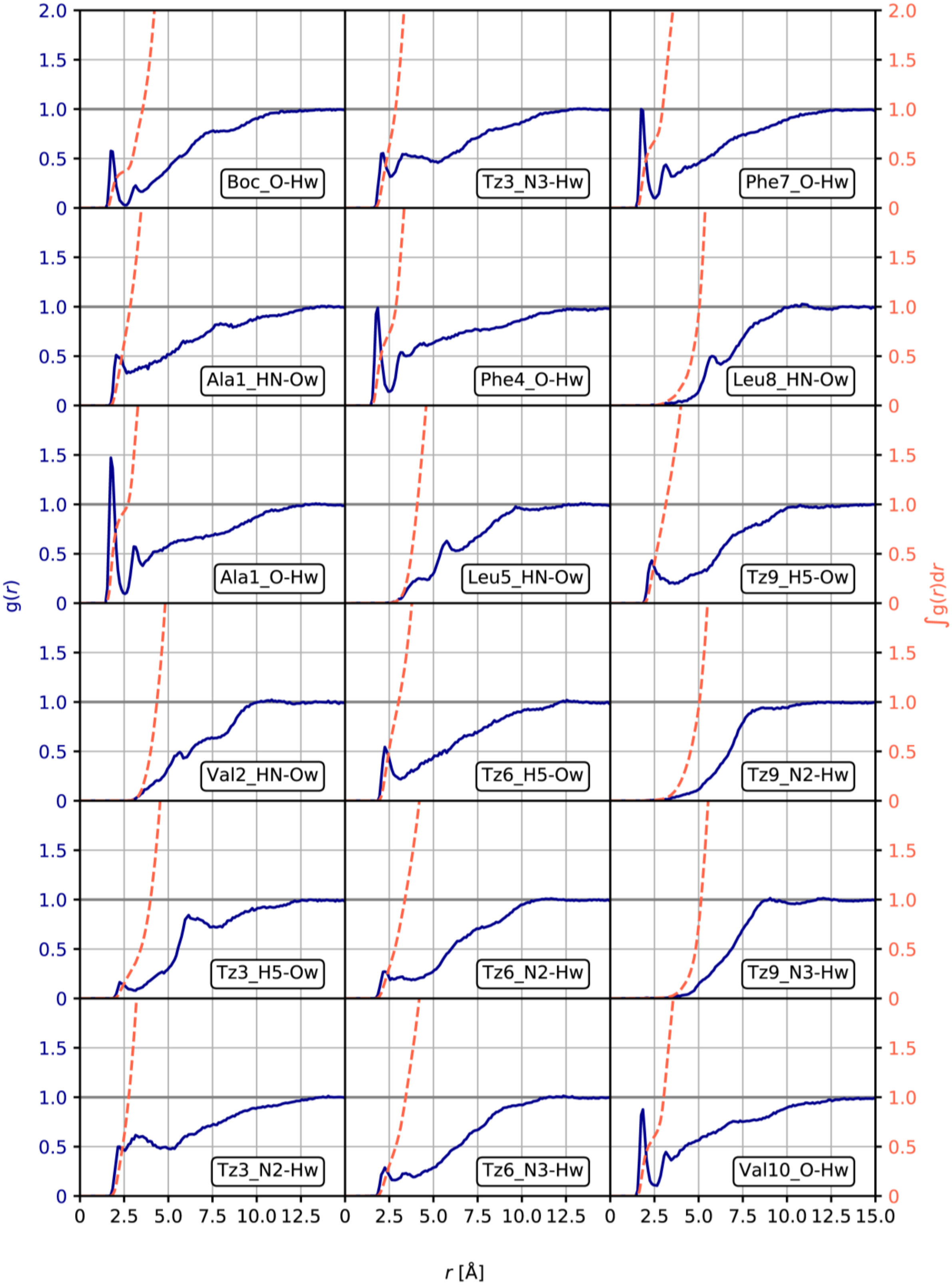

Supplementary Figure 12. Radial distribution functions for chosen backbone atoms of the homochiral oligomer.

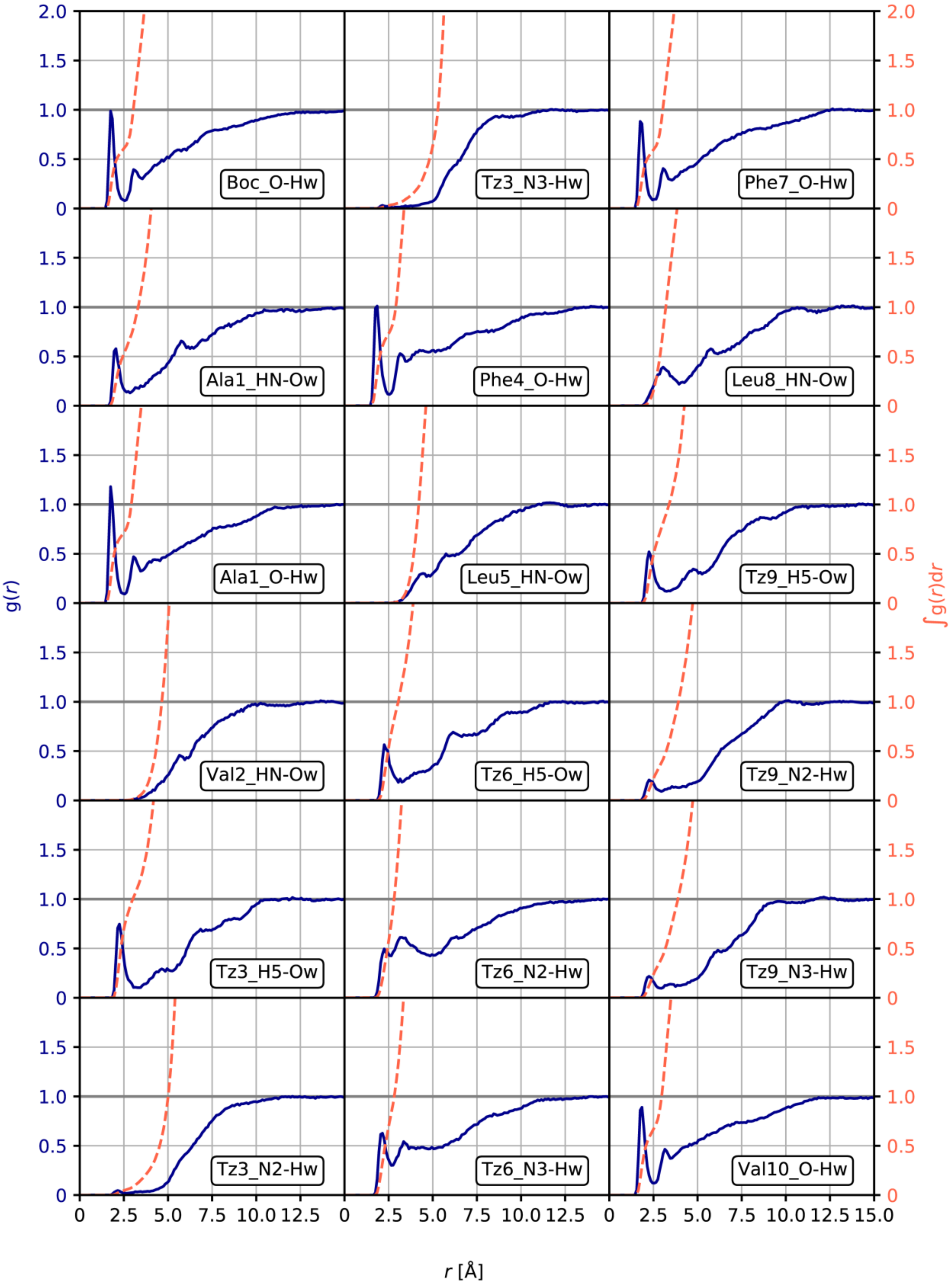

Supplementary Figure 13. Radial distribution functions for chosen backbone atoms of the heterochiral oligomer.

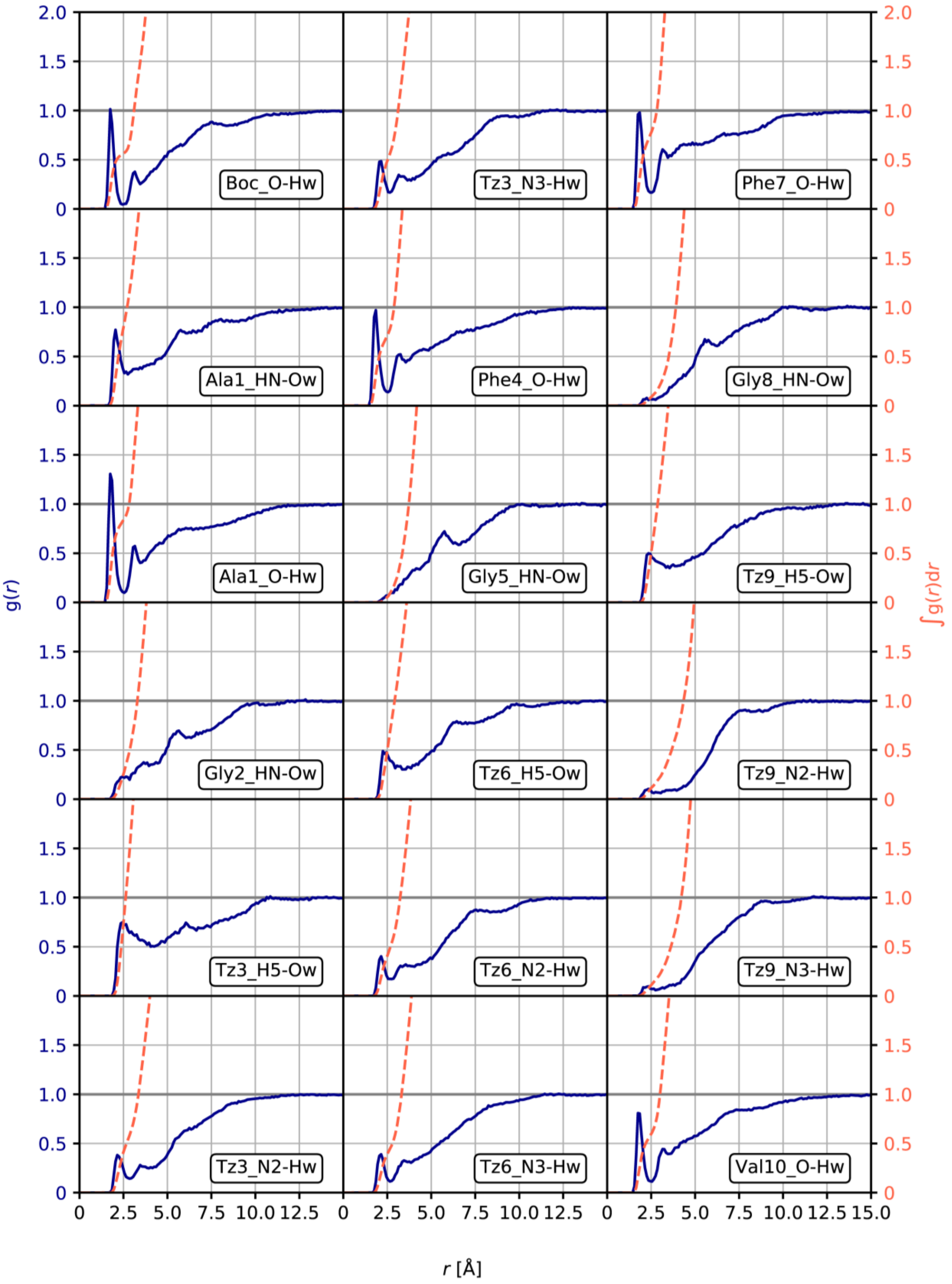

Supplementary Figure 14. Radial distribution functions for chosen backbone atoms of the homochiral* oligomer.

2.2 Homochiral, heterochiral, and homochiral* poly-alanine derivatives

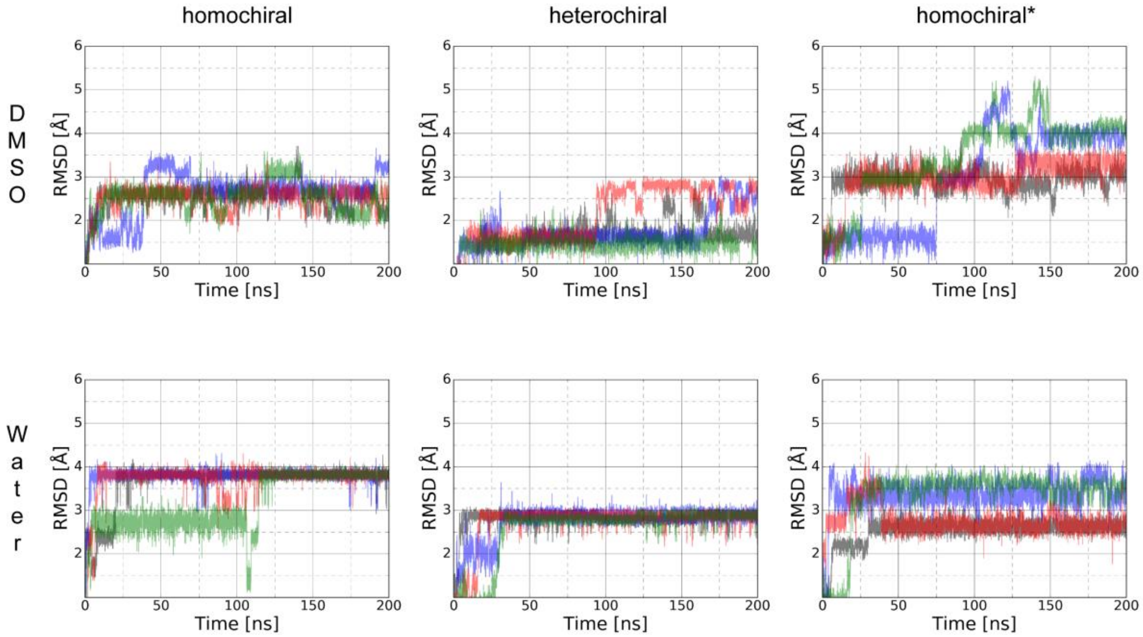

Supplementary Figure 15. Backbone radius of gyration time series for the whole trajectory of homochiral, heterochiral, and homochiral* poly-alanine derivatives in DMSO (first row) and TIP4Pew water (second row).

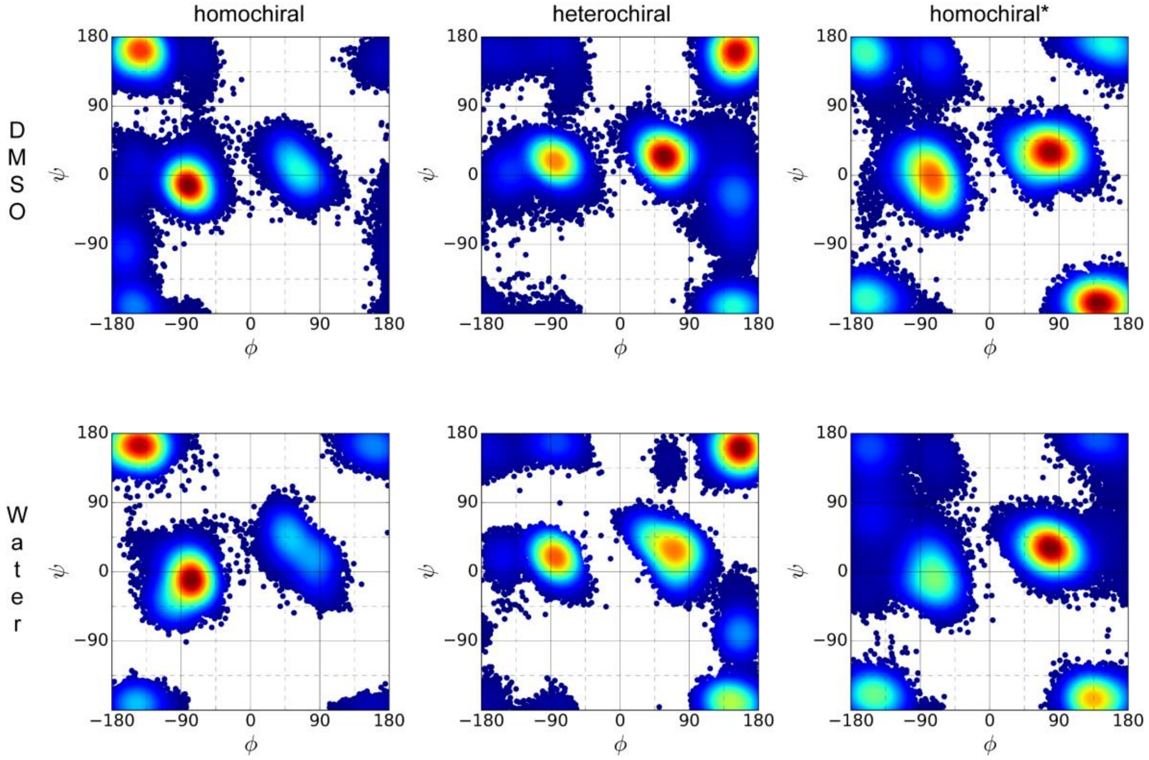

Supplementary Figure 16. φ/ψ torsion angles distribution of homochiral, heterochiral, and homochiral* poly-alanine derivatives for the last 100 ns of molecular dynamics in DMSO (first row) and TIP4Pew water (second row), merged over 4 independent trajectories each.
